# Descriptions of sham acupuncture in randomised controlled trials: a critical review of the literature

**DOI:** 10.1186/s12906-023-04007-7

**Published:** 2023-05-30

**Authors:** Yixuan Xie, Xiaoyu Liu, Tinglan Liu, Chiyun Sun, Zeyin Xin, Yuzhi Hu, Yue Wang, Cheng Zhang, Shiyan Yan

**Affiliations:** 1 School of Acupuncture-Moxibustion and Tuina, Beijing University of Chinese Medicine, Beijing, China; 2 School of Acupuncture-Moxibustion and Tuina, Tianjin University of Traditional Chinese Medicine, Tianjin, China

**Keywords:** Sham acupuncture, Reporting quality, RCTs, STRICTA 2010, TIDieR-Placebo

## Abstract

**Background:**

Sham acupuncture is usually used to assess the specific effects of acupuncture. However, the reporting quality of sham acupuncture remains unclear despite its critical importance in understanding and analyzing the effects of acupuncture. This paper presents a literature review aimed at assessing the quality of reporting of sham acupuncture in randomized controlled trials (RCTs) based on STRICTA 2010 and TIDieR-Placebo.

**Methods:**

Three electronic English-language databases (PubMed, MEDLINE and Embase) were searched from inception to March 7, 2022, and RCTs of sham acupuncture were identified. The reporting quality of sham acupuncture was assessed in accordance with the items recommended in STRICTA 2010 and TIDieR-Placebo. The reporting quality of other items related to sham acupuncture apart from items from these two checklists was also captured to further assess the reporting quality of sham acupuncture.

**Results:**

A total of 609 eligible studies were included. For all of the items recommended in STRICTA 2010 and TIDieR-Placebo, 100% of the studies reported a brief name that described the sham acupuncture, 93.9% studies reported the needle type, and 90.0% reported the names of the points used. Other items for which the reporting rates were above 50% included the number, frequency and duration of treatment sessions; needle retention time; and number of needle insertions per subject per session. Overall, 49.4% of the studies revealed the rationale why sham acupuncture was chosen, 39.7% of the studies involving insertion processes reported the depth of insertion, and 37.9% of the studies reported the needle manufacturer. Other items for which the reporting rates were below 30% included practitioner-related information, response sought, evaluation of blinding, intervention mode and environment, assisting tools, and the extent to which the treatment was varied. The items “Modifications”, “How well (planned)” and “How well (actual)” were not reported in any of the analyzed studies.

**Conclusions:**

The overall reporting quality of sham acupuncture in RCTs was suboptimal. Although STRICTA 2010 and TIDieR-Placebo could be beneficial for describing sham acupuncture, neither can offer recommendations specifically for sham acupuncture. There is thus an urgent need to develop specialized guidelines for reporting sham acupuncture in clinical trials.

**Supplementary Information:**

The online version contains supplementary material available at 10.1186/s12906-023-04007-7.

## Background

Reporting the quality of interventions has been a focus of researchers in order to better interpret and analyze clinical trials of acupuncture. Efforts to improve descriptions of acupuncture interventions have continuously been made. Standards for Reporting Interventions in Controlled Trials of Acupuncture (STRICTA 2010 [1]), as an official extension of CONSORT [2], comprising initiative guidelines with recommendations aimed at more adequately reporting interventions, has long been applied for guiding descriptions of acupuncture interventions in clinical trials. It is intended to facilitate better understanding of trial design and conduct, critical appraisal, analysis and replication of acupuncture clinical trials.

Sham-controlled trials have been widely used in clinical trials of acupuncture [3–5]. Sham acupuncture, a comparison control for assessing the specific effect of real acupuncture, has been applied in several forms, including nonpenetrating needling, shallow needling on points or non-points, regular needling on non-points [6], and sham manipulation with various devices [7, 8]. The effects of different placebos vary [9–13], and sham acupuncture has greater effects than other placebos [12, 14]. Zeng [6] found high variability in placebo responses in different forms of sham acupuncture. A better understanding of sham acupuncture may facilitate the interpretation of research results, leading to a more rigorous study design to promote healthcare policies and practice in the future. However, despite the long history of researchers focusing on descriptions of acupuncture interventions [15–19], the reporting quality of sham acupuncture remains unclear.

Existing published guidelines, such as STRICTA 2010 [1] and TIDieR-Placebo [20], may help us to appraise the quality of reporting of sham acupuncture to a certain degree. STRICTA 2010 (containing 6 items with 17 sub-items) recommends “Precise description of the control or comparator. If sham acupuncture or any other type of acupuncture-like control is used, provide details as for items 1–3”, namely, “Acupuncture rationale” “Details of needling” and “Treatment regimen”. The TIDieR-Placebo checklist is a set of guidelines specifically developed to encourage precise and accurate reporting of the nature and implementation of placebo/sham interventions. The items recommended in these two sets of guidelines share common considerations of sham controls, while they vary in the way in which different types of sham interventions are covered. To better understand the transparency of reporting of sham acupuncture in RCTs, the combined use of these two guidelines is necessary.

This review aims to comprehensively evaluate the reporting quality of sham interventions in RCTs of acupuncture based on STRICTA 2010 and TIDieR-Placebo, and to discuss the necessity of developing a new set of guidelines for reporting sham acupuncture.

## Methods

### Search strategy

The following electronic English-language databases were searched from inception to March 7, 2022, for RCTs of sham acupuncture: PubMed, MEDLINE and Embase. Our search strategies were iteratively developed using the terms “acupuncture”, “sham” and “randomized controlled trial” along with synonyms of these latter two terms (see Supplementary File 1 for the complete search strategy). The language of the publications was restricted to English and the subjects of the studies were restricted to humans. In addition, a search of further potentially relevant papers was conducted in the references listed in the identified papers.

### Inclusion and exclusion criteria

RCTs involving sham acupuncture as a control intervention were included. Sham acupuncture refers to sham manual acupuncture, sham electroacupuncture, sham laser acupuncture, and sham transcutaneous electrical nerve stimulation (TENS)/sham transcutaneous electrical acupoint stimulation (TEAS). Guidelines, reviews, systematic reviews, meta-analyses, case reports, conference papers, animal experiments, randomized controlled crossover trials, pilot trials, academic theses, and studies assessing the sham needle effect or studies of which the subjects were healthy volunteers were excluded. RCTs with sham interventions mimicking acupoint injection, massage, acupressure, moxibustion, acupoint application, acupoint embedding, auricular acupuncture or acupressure, cupping, scraping, scalp acupuncture, abdominal acupuncture, and other unrelated forms of interventions were also excluded. RCTs for which the full text of the associated report was not available were excluded from this study.

### Data extraction and analysis

After a basic literature search, titles and abstracts were screened for eligibility and full texts of reports on potentially eligible studies were retrieved and their eligibility was confirmed. We used EndNote20.5 for the screening and data extraction processes. Two reviewers (Liu XY and Xie YX) independently conducted the screening process and their results were crosschecked against each other. Any disagreement was resolved by discussion. A predesigned standardized Excel sheet was used to load all of the information about sham acupuncture based on the corresponding items recommended in STRICTA 2010 and TIDieR-Placebo as well as other relevant items (established by consultations with experts). After uniform training and pilot extraction with examination, six reviewers (Hu YZ, Liu TL, Sun CY, Wang Y, Xin ZY, and Zhang C) were divided into two groups and completed the extraction of literature information. Members of each group independently extracted information from half of the literature and then crosschecked their information with that extracted by the their counterparts; differences were resolved via discussion with Liu XY and Xie YX until an agreement was reached. After reading all of the obtained literature, it was determined whether the relevant item was reported. In the corresponding tables of the Excel sheet, “N” indicates that the item was not reported, while “Y” indicates that it was reported.

## Results

**Fig. 1:**
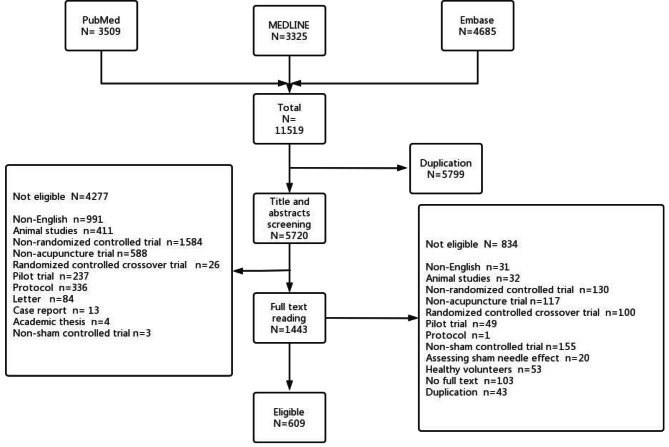
Study flow diagram

After a basic search, 11,519 reports were collected. After the removal of 5799 duplicates, 5720 studies remained after the first round of screening. By screening of the titles and abstracts, a further 4277 papers were excluded. With 1443 potentially eligible studies left, another 834 papers were further excluded through screening of the full texts, which left 609 eligible studies for final inclusion in this review (Fig. 1). The items of sham acupuncture reported in all included reports are listed in tables in Supplementary File 2 with references in Supplementary File 3.

### Reporting quality based on STRICTA 2010 and TIDieR-Placebo

Table 1 shows the reporting quality of similar items both recommended in the two checklists. For the item “Acupuncture rationale”, the sub-item “Style of acupuncture” was not relevant to the reporting of sham acupuncture. In addition, 49.4% of the studies provided the rationale for choosing sham acupuncture and 4.9% described the extent to which sham treatment varied. For the item “Details of needling”, 53.7% of studies described the number of needle insertions per subject per session and 90.0% studies reported the names of points used in the sham group. Of the 441 trials involving needle insertion, 39.7% studies reported insertion depth and 20.6% reported whether the trial described deqi sensation in the sham acupuncture control. Furthermore, needle retention time was reported in 65.4% of the available studies. For the sub-item “Needle stimulation”, 41.5% of studies were found to have reported it. More details of the reporting conditions for needle stimulation are as follows: Of the 441 trials featuring needle insertion (including sham manual acupuncture and sham electroacupuncture), 7.3% reported basic insertion techniques including lifting, thrusting or rotating the needle; 3.4% reported the frequency of needling manipulation; 3.9% reported the time point of manipulation; 5.7% reported the times of needling manipulation; 3.4% reported the duration of each manipulation; and no trials reported any assisting needling technique. Among 305 trials involving electrical stimulation (including sham electroacupuncture, laser acupuncture, and sham TENS/TEAS), 64.9% reported the intensity of electrical stimulation, 45.9% reported the frequency of electrical stimulation, 15.1% reported the electrical stimulation waveform, and 16.4% reported the wave width. Additionally, the sub-item “Needle type” in STRICTA 2010 recommended reporting types of needles used, including the diameter, length, manufacturer, and/or material. We found that the rates of reporting needle type, needle size, assisting tools, and manufacturer of needles were 93.9%, 44.3%, 14.4%, and 37.9%, respectively. For the item “Treatment regimen”, 77.8% of the papers reported the number of treatment sessions, 75.9% reported the frequency of treatment sessions, and 78.0% reported the duration of treatment sessions.

**Table 1:** Sham-acupuncture related items recommended in both STRICTA 2010 and TIDieR-placebo

Items in STRICTA 2010		Items in TIDieR-placebo	Proportion
Acupuncture rationale	(1a) Style of acupuncture (e.g., Traditional Chinese Medicine, Japanese, Korean, Western medical, Five Element, ear acupuncture, etc.)		NR
	(1b) Reasoning for treatment provided, based on historical context, literature sources and/or consensus methods, with references where appropriate	2.Why: Describe any rationale, theory, or goal of the elements essential to the placebo/ sham intervention	49.4%
	(1c) Extent to which treatment was varied	9.Tailoring: If the placebo/sham intervention was planned to be personalised, titrated, or adapted, then describe what, why, when, and how	4.9%
Details of needling	2a) Number of needle insertions per subject per session (mean and range where relevant)	4. What (Procedure): Describe each of the procedures, activities, and/or processes used in the placebo/sham intervention, including any enabling or support activities	53.7%
	2b) Names (or location if no standard name) of points used (uni/bilateral)		90.0%
	2c) Depth of insertion, based on a specified unit of measurement, or on a particular tissue level		39.7%
	2d) Response sought (e.g. de qi or muscle twitch response)		20.6%
	2e) Needle stimulation (e.g. manual, electrical)		41.5%
	2f) Needle retention time		65.4%
	2 g) Needle type (diameter, length, and manufacturer or material)	3.What (materials): Describe any physical or informational materials used in the placebo/sham intervention, including those provided to participants or used in intervention delivery or in training of intervention providers. Provide information on where the materials can be accessed (such as an online appendix, URL)	93.9%(type)
			44.3%(size)
			14.4%(assisting tools)
			37.9%(manufacturer)
Treatment regimen	(3a) Number of treatment sessions	8.When and how much: Describe the number of times the placebo/sham intervention was delivered and over what period of time, including the number of sessions, their schedule, and their duration, intensity, or dose. If relevant, include the duration of the pre- and postrandomisation consultations	77.8%
	(3b) Frequency and duration of treatment sessions		75.9%
			78.0%

NR = Not relevant

### Quality of reporting of items only recommended in TIDieR-Placebo

Eight items only recommended in TIDieR-Placebo are listed with reporting rates in Table 2, namely, “Brief name”, “Who provided”, “How”, “Where”, “Modifications”, “How well (planned)”, “How well (actual)” and “Measuring the success of blinding”. Among 609 trials, 100% of the papers offered a brief name or phrase that described sham acupuncture. As for the practitioners, 23.3% of trials reported the practitioners’ working experience, 28.6% reported the practitioners’ educational background, and 12.5% and 35.6% described the practitioners’ training and profession, respectively. Moreover, 10.0% and 3.8% of trials reported the intervention mode and intervention environment of the sham acupuncture, respectively. For the items “Modifications”, “How well (planned)”, and “How well (actual)”, no available studies reported related contents. In terms of blinding evaluation, 15.8% (96 studies) of trials reported whether the trial involved one blinding evaluation or more. However, among the 96 studies that reported blinding evaluation, 94.8% of them reported the evaluated subjects, 92.7% reported the number of evaluations, 89.6% reported the time point of evaluation, 86.5% reported the evaluation questions, 95.8% reported the evaluation results, and 96.9% reported the indicators of successful blinding.

**Table 2:** Items only recommended in TIDieR-Placebo

Items	Proportion
1. Brief name: Provide the name or a phrase that describes the placebo/sham intervention	100%
5.Who provided: For each category of placebo/sham intervention provider (such as psychologist, nursing assistant), describe their expertise, background, and any specific training given	23.3%(Work experience)
	28.6%(Educational background)
	12.5%(Training)
	35.6%(Profession)
6.How: Describe the modes of delivery (such as face to face or by some other mechanism, such as internet or telephone) of the intervention and whether it was provided individually or in a group	10.0%
7.Where: Describe the type(s) of locations(s) and settings where the placebo/sham intervention occurred, including any necessary infrastructure or relevant features	3.8%
10.Modifications: If the placebo/sham intervention was modified during the course of the study, describe the changes (what, why, when, and how)	0
11. How well (planned): If placebo/sham intervention adherence or fidelity was assessed, describe how and by whom, and if any strategies were used to maintain or improve fidelity, describe them	0
12.How well (actual): If placebo/sham intervention adherence or fidelity was assessed, describe the extent to which the intervention was delivered as planned	0
13.Measuring the success of blinding: Was blinding measured, and if so, how, and what were the results of such measurement	15.8%

### Other related extracted items

Apart from items included in these two checklists, we collected reported data for other items. As shown in Table 3, among 441 trials involving the insertion process, 1.6% reported the insertion method, 5.2%reported the insertion angle, 2.7% reported the insertion direction, 1.1% reported the insertion order, and 11.1% reported needle removal. Of the 305 trials using electrical equipment (including sham laser acupuncture, sham electroacupuncture and sham TENS/TEAS), 56.1% reported the model of such equipment.

**Table 3:** Other items related to sham acupuncture controls

Reporting items	Numerator	Denominator	Reporting proportion
Model of electrical equipment*	171	305	56.1%
Needle removing method**	49	441	11.1%
Intervention information offered to patients***	104	609	17.1%
Patient body position***	92	609	15.1%
Communication with participants before intervention***	80	609	13.1%
Disinfection***	70	609	11.5%
Insertion angle**	23	441	5.2%
Communication with participants during intervention***	25	609	4.1%
Communication with participants after intervention***	25	609	4.1%
Insertion direction**	12	441	2.7%
Insertion order**	5	441	1.1%
Information offered in informed consent***	11	609	1.8%
Insertion method**	7	441	1.6%
Operation before needle insertion(stimulation)***	9	609	1.5%
Operation after needle removing(stimulation)***	5	609	0.8%

* denoting items involving interventions including sham electroacupuncture, sham laser acupuncture and TENS/TEAS

** denoting items involving interventions including sham manual acupuncture and sham electroacupuncture

***denoting items involving all sham acupuncture included in this study

Meanwhile, we found that the disinfection procedure was reported in 11.5% of studies. Among these, 28.6% reported the disinfection of needles, while 81.4% reported the disinfection materials. Patient’s body position was reported in 15.1% of trials. Information about sham acupuncture for which informed consent was obtained was provided for 1.8% of all 609 trials; meanwhile, 17.1% of trials reported information about the intervention offered to patients. Overall, 13.1% of trials reported communication before intervention, 4.1% of trials reported communication during intervention, and 4.1% reported communication after intervention. Only 1.5% of the papers reported any operation before needle insertion, and 0.8% reported an operation after needle removal.

### Descriptions of sham laser acupuncture and sham TENS/TEAS

Considering the specific characteristics of sham laser acupuncture and sham TENS/TEAS, data of descriptions of these procedures were analyzed separately based on items recommended in STRICTA 2010 and TIDieR-Placebo, as well as other related items (see Supplementary File 4 for details).

In general, the reporting quality of sham laser acupuncture and TENS/TEAS did not differ much from that of all included trials, except that sub-items such as “Needle type”, practitioner-related information, model of electrical equipment, and blinding evaluation were found to have lower reporting rates, while the sub-item “Needle stimulation” was found to have a higher one.

## Discussion

Our study comprehensively reviewed the reporting quality of sham acupuncture in relevant RCTs based on STRICTA 2010 and TIDieR-Placebo. The overall reporting quality of sham acupuncture was suboptimal. Items for which the reporting rate was above 50% included a brief name that described the sham acupuncture, needle type, names of points used, number, frequency, and duration of treatment sessions, needle retention time, and number of needle insertions per subject per session. Items for which the reporting rate was between 30% and 50% included the rationale for choosing sham acupuncture, needle stimulation, depth of insertion, and the manufacturer of the needles. Other items for which the reporting rate was below 30% included practitioner-related information, response sought, measurement of blinding, intervention mode and environment, assisting tools, and the extent to which treatment varied. Items including “Modifications”, “How well (planned)” and “How well (actual)” were not found to be reported in any of the literature obtained.

Our results were consistent with those of a previous study. A cross-sectional evaluation [21] of sham acupuncture descriptions revealed that between 2009 and 2018, the reporting qualities of sham acupuncture remained low and did not improve over time, only 7 items out of 25 were found to be reported in more than 50% of the studies obtained. Another similarity is that both studies found that the number of sham needles, needle retention time, locations of points used, and treatment sessions were highly reported items, whereas items concerning the categories of “practitioner” and “protocol and settings” [21] were seldom reported. However, there are subtle differences between the previous study and our study. For example, the item “depth of insertion” was found to be reported in over 50% of studies in the previous study, but in less than 50% of the available studies in our study. Meanwhile, the item “needle stimulation” was reported in less than 50% of studies in the previous study but in more than 50% of available studies concerning sham laser acupuncture and TENS/TEAS (not all studies included) in our study. In addition, the previous study did not capture the quality of descriptions of important elements of sham acupuncture, such as needle model, assisting tools, manufacturer of needles, brief name of sham acupuncture, intervention mode and environment, and practitioner background. Differences in sample size and in the guidelines of the previous study and ours might partially explain these distinct results. The previous study included 117 studies for analysis since it only involed a search of the PubMed database for articles published from 2009 to 2018, and excluded trials using sham interventions such as sham electroacupuncture, sham TENS/TEAS, and sham laser acupuncture. In our study, three databases were searched for articles published from inception to 2022 and included trials using multiple kinds of sham acupuncture, thus having a larger sample size. Besides, items for evaluation in the previous study were determined according to STRICTA, CONSORT and TIDieR [22], however, in our study, items were chosen based on STRICTA 2010 and TIDieR-Placebo which was specially designed for sham interventions including but not limited to sham acupuncture, and other considerations.

We found that, for most items extracted in this study, compared with the overall descriptions of sham acupuncture, the reporting rates of items of sham laser acupuncture and sham TENS/TEAS did not seem to differ markedly. However, some items, showed subtle differences. Lower reporting rates were shown in the item of practitioner-related information, including work experience, educational background, training, and profession. We infer that the inadequacy of descriptions of practitioners might be mainly due to the lower dependence on practitioners’ skills in sham laser acupuncture and TENS/TEAS than in sham manual acupuncture and sham electroacupuncture. Therefore, researchers may not pay sufficient attention to describing this information. Sub-items such as “Needle type”, blinding evaluation, and model of electrical equipment also had lower reporting rates than those of all trials. This information needs more attention from researchers. Meanwhile, the sub-item “Needle stimulation”, which refers to the settings of electrical stimulation including intensity, frequency, waveform, and width, was found to have a higher reporting rate than the overall level. Parameters of electric stimulation are key factors of sham laser acupuncture and sham TENS/TEAS, so reporters paid more attention to describing these contents.

Considering the complexity of sham acupuncture components, the effects of sham acupuncture are largely influenced by specific design details. Contextual factors [23], such as treatment settings, intervention mode and environment, practitioner experience, practitioner-patient relationship, and patients’ understanding of or expectations toward acupuncture intervention [24–26], are considered to be among the main contributors to the effectiveness of acupuncture. Altogether, contextual factors along with specific details of the sham acupuncture design can have a nonspecific (potentially specific) yet statistically and clinically significant influences on outcomes [27–29]. However, STRICTA 2010 recommendations showed the significance of specific components of needling while neglecting many of the contextual factors. Regarding contextual factors in sham controls, several recommendations listed in TIDieR-Placebo, although not specifically designed for sham acupuncture, could be combined with sham acupuncture settings to guide the reporting of contextual factors in sham acupuncture. For example, considering the possible influence of practitioner-related characteristics on outcomes, STRICTA 2010 recommended that researchers report the relevant background of practitioners who implement the interventions, such as their qualifications or profession years spent practicing acupuncture and other relevant experience. When guiding the reporting of sham acupuncture, relevant information from practitioners in the sham acupuncture group was not required to be reported. Regardless of its low reporting quality, practitioner background was required in item 5 “Who provided” of TIDieR-Placebo. In addition, more contextual factors such as communication with participants, information offered in informed consent, and intervention information offered to patients might affect patients’ psychological state and expectations [30], influencing research results and necessitating transparent descriptions for better interpretation.

Besides contextual factors, to better comprehend the effects of sham acupuncture, other important factors such as modifications of the sham acupuncture regimen, practitioner adherence, and blinding evaluation need more consideration. Owing to the complex components and manipulations of sham acupuncture, unforeseen modifications might occur during actual clinical trials. Modifications of sham acupuncture might lead to altered procedures or circumstances of implementation [20]. The reporting of any modification of the sham acupuncture procedure or implementation is warranted for trial transparency and better interpretation and replication. Another factor that has long been neglected in reporting is practitioner adherence, which refers to whether the practitioner performs each step of the sham acupuncture as planned. It is crucial to accurately assess and monitor whether practitioners’ practices meet the predefined requirements and whether planned sham acupuncture protocols and procedures are consistently performed as different effects may occur due to differences in stimulation parameters, such as stimulated points and insertion depth. Regarding blinding evaluation, comprehensively assessing and reporting the results of the blinding status can provide readers with more evidence about the validity and credibility of sham acupuncture in the trial. Although few relevant studies were found to report these items, they should still be emphasized as they can significantly alter outcomes.

After STRICTA 2010 was published, researchers suggested that some important elements, such as the angle and direction of insertion [31], subjects’ prior experiences of acupuncture, as well as standards and methods on acupoint selection and location [32], should be considered in STRICTA 2010, considering their potential to impact the effects of acupuncture. Items beyond STRICTA 2010 and TIDieR-Placebo, such as insertion method and patient’s body position, are also important factors that might influence the effects of acupuncture and are seldom adequately reported. Lack of this critical information may lead to ambiguity for readers to comprehend the real effects of sham acupuncture, thus leading to overestimation or underestimation of the specific effects of acupuncture. With regard to their impact on the effects of sham acupuncture, we strongly argue that these items should be reported in the RCT literature with regard to sham acupuncture.

It was assumed that the low reporting rate of sham acupuncture items might be partly due to the lack of specific guidelines for promoting the reporting of sham acupuncture. Currently, STRICTA 2010 and TIDieR-Placebo are adopted in combination to examine the quality of reporting of sham acupuncture. However, neither of them was specifically developed for this purpose. In general, STRICTA 2010 tends to be more relevant to acupuncture intervention, while TIDieR-Placebo generally covers guidance toward a wider range of sham interventions. For example, item 4 “What (Procedure)” of the TIDieR-Placebo refers to a requirement “Describe each of the procedures, activities, and/or processes used in the placebo/sham intervention, including any enabling or support activities.” Correspondingly, items in STRICTA 2010 include “Number of needle insertions per subject per session”, “Names of points used”, “Depth of insertion”, “Response sought”, “Needle stimulation” and “Needle retention time”. Moreover, when used as a guiding/evaluation tool, both of them tend to fail in terms of adequacy and accuracy; i.e., both checklists lack adequate guidance toward the specific reporting of sham acupuncture. Our previous study also found that both checklists could provide certain references for descriptions of sham acupuncture, but neither seemed to be fully applicable after performing an in-depth comparison of the STRICTA 2010 and TIDieR-Placebo [33]. Hence, we strongly recommend the development of a specialized set of guidelines for guiding the reporting of sham acupuncture in sham-controlled trials of acupuncture based on STRICTA 2010 and TIDieR-Placebo. As the Acupuncture Controls gUideline for Reporting humAn Trials and Experiments (ACURATE) checklist [34] got published, efforts of clear descriptions of sham acupuncture in accordance with ACURATE checklist should be made to facilitate interpretation and replication of trials and precisely assess specific effects of acupuncture.

Our study has several strengths. First, this work involved a comprehensive literature review with a large sample size to assess the quality of reporting of sham acupuncture in RCTs. To identify as many eligible trials as possible, we comprehensively searched the three most commonly used databases (PubMed, MEDLINE and Embase) from inception to 2022 to obtain a substantial amount of literature. This is also the first study to analyze the quality of reporting of sham acupuncture based on STRICTA 2010 and TIDieR-Placebo, as well as other important items related to sham acupuncture settings. Our study provides validated evidence for the quality of reporting of sham acupuncture and is critical to encouraging researchers to pay more attention to improving the transparency of sham acupuncture. However, this study has some limitations. First, we only included studies reported in English based on the consideration that most sham-controlled RCTs were reported in English. Besides, we may have made a subjective judgment when determining if items recommended in STRICTA 2010 and TIDieR-Placebo were reported in available literature because one item may contain information from several aspects, and partially reported items were categorized as “N”. We also did not evaluate the overall quality of the included studies, which might have influenced the strength of our results.

## Conclusions

The overall reporting quality of sham acupuncture in relevant RCTs was rather suboptimal. Clear descriptions of sham acupuncture in clinical trials are necessary. ACURATE checklist, a reporting guideline for sham acupuncture, should be encouraged to be used.

## Electronic supplementary material

Below is the link to the electronic supplementary material.

Supplementary Material 1

Supplementary Material 2

Supplementary Material 3

Supplementary Material 4

## Data Availability

The datasets used and/or analysed during the current study are available from the corresponding author on reasonable request.

## Abbreviations

RCT: Randomized controlled trials
STRICTA: Standards for Reporting Interventions in Controlled Trials of Acupuncture
TENS: Transcutaneous Electrical Nerve Stimulation
TEAS: Transcutaneous Electrical Acupoint Stimulation
