# Supplementary material for: Descriptions of sham acupuncture in randomised controlled trials: a critical review of the literature
Source: BMC Complement Med Ther. 2023 May 30;23:173. doi: 10.1186/s12906-023-04007-7 (PMC10227975; doi:10.1186/s12906-023-04007-7)
Supplement: Supplementary file 1 — Supplementary Material 1 [file 12906_2023_4007_MOESM1_ESM.docx]

Search time: 2022/03/07

Total studies：11519

**PubMed 3509**

**#1：**

("Controlled Clinical Trial" [Publication Type]) OR "Randomized Controlled Trial" [Publication Type]OR"Controlled Clinical Trials as Topic"[Mesh] OR "Clinical Trials as Topic" [Mesh] OR "Randomized Controlled Trials as Topic" [Mesh] OR Clinical Trial [Title/Abstract] OR Controlled Clinical Trial [Title/Abstract] OR Randomized Controlled Trial [Title/Abstract] OR randomized [Title/Abstract] OR randomly [Title/Abstract] OR trial[Title/Abstract]

#2：

"Acupuncture"[Mesh] OR "Acupuncture Therapy"[Mesh] OR "Acupuncture Analgesia"[Mesh] OR "Electroacupuncture"[Mesh] OR "Meridians"[Mesh] OR "Acupuncture Points"[Mesh] OR acupuncture[Title/Abstract] OR acupoint[Title/Abstract] OR acupuncture point[Title/Abstract]OR electroacupuncture[Title/Abstract] OR electro-acupuncture[Title/Abstract]

#3：

("placebos"[MeSH Terms] OR "sham"[Title/Abstract] OR "placebo"[Title/Abstract] OR "fake"[Title/Abstract] OR "false"[Title/Abstract] OR "simulated"[Title/Abstract] OR "imitation"[Title/Abstract] OR "mock"[Title/Abstract])


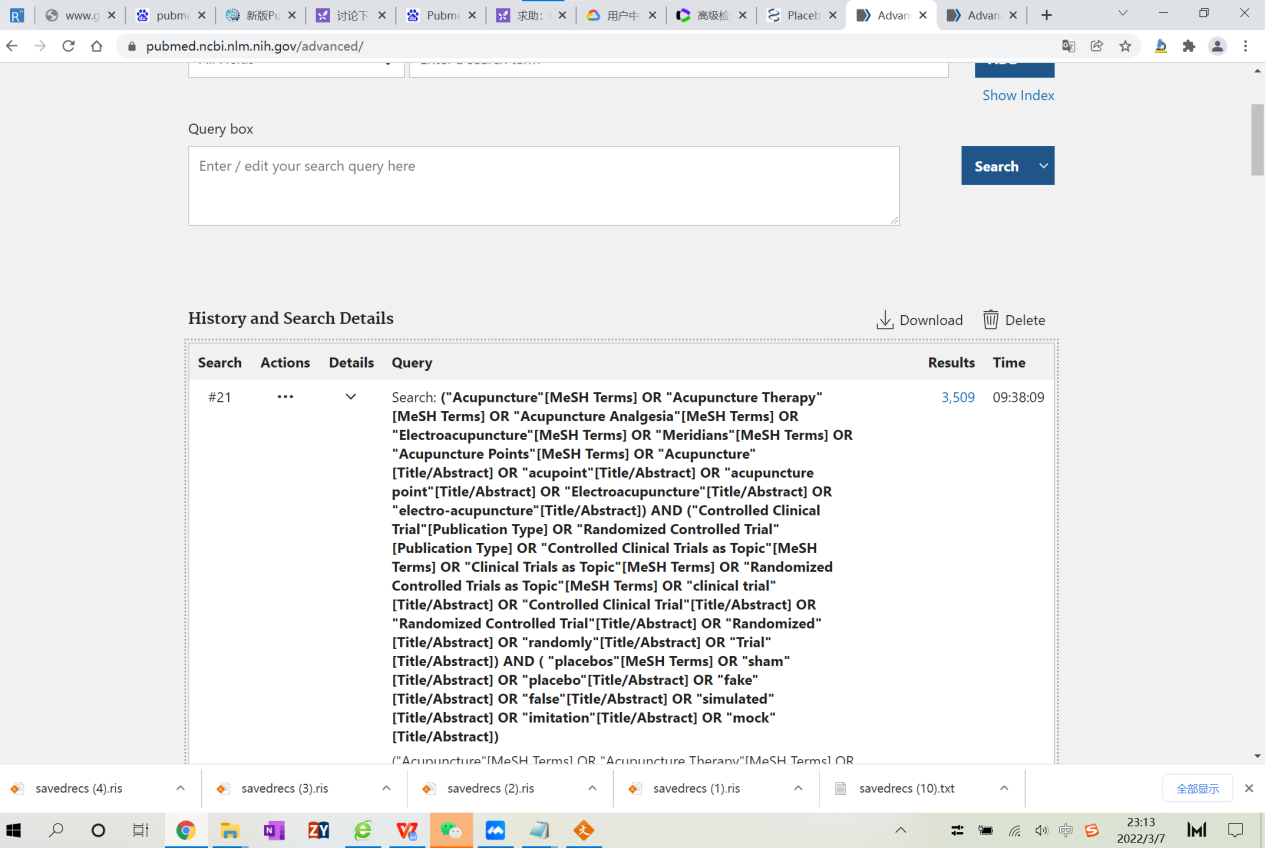


**MEDLINE 3325**

Link:

https://www.webofscience.com/wos/medline/summary/b72e8dbc-a8ce-4384-b75c-8a3157a3eb9a-2830d767/relevance/1

#1

((((((((MHX=(randomized controlled trial)) OR DT=(Controlled Clinical Trial)) OR DT=(Randomized Controlled Trial)) OR AB=(Clinical Trial)) OR AB=(controlled clinical trial)) OR AB=( randomized controlled trial)) OR AB=(randomized)) OR AB=( randomly)) OR AB=(trial)
#2

((((((((MHX=(acupuncture)) OR AB=(acupuncture)) OR AB=(acupuncture point)) OR AB=(acupuncture points)) OR AB=(acupoint)) OR AB=(acupoints)) OR AB=(electroacupuncture)) OR AB=(electro acupuncture)) OR AB=(electro-acupuncture)

#3

(((((((MHX=(placebo)) OR AB=(placebo)) OR AB=(sham)) OR AB=(fake)) OR AB=(false)) OR AB=(mock)) OR AB=(simulated)) OR AB=(imitation)


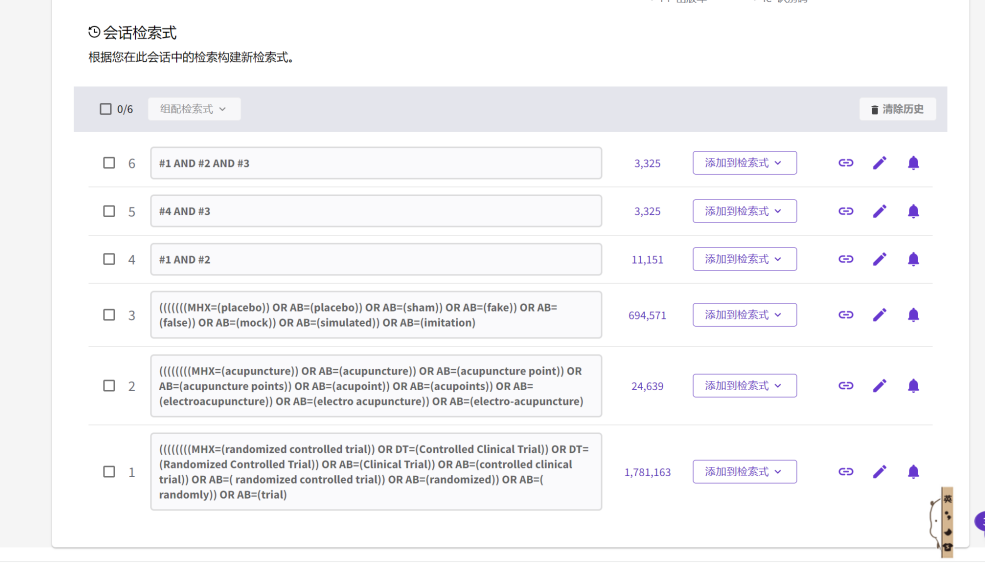


Embase 4685


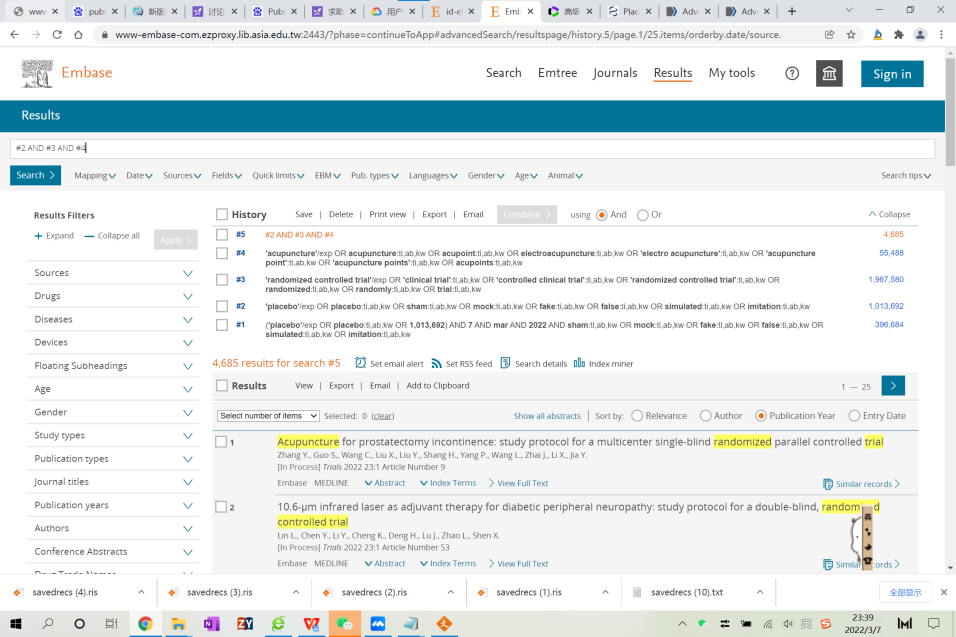


## Embase Session Results (7 Mar 2022)

| No. | Query | Results |
| --- | --- | --- |
| #5 | #2 AND #3 AND #4 | **4685** |
| #4 | 'acupuncture'/exp OR acupuncture:ti,ab,kw OR acupoint:ti,ab,kw OR electroacupuncture:ti,ab,kw OR 'electro acupuncture':ti,ab,kw OR 'acupuncture point':ti,ab,kw OR 'acupuncture points':ti,ab,kw OR acupoints:ti,ab,kw | **55488** |
| #3 | 'randomized controlled trial'/exp OR 'clinical trial':ti,ab,kw OR 'controlled clinical trial':ti,ab,kw OR 'randomized controlled trial':ti,ab,kw OR randomized:ti,ab,kw OR randomly:ti,ab,kw OR trial:ti,ab,kw | **1967580** |
| #2 | 'placebo'/exp OR placebo:ti,ab,kw OR sham:ti,ab,kw OR mock:ti,ab,kw OR fake:ti,ab,kw OR false:ti,ab,kw OR simulated:ti,ab,kw OR imitation:ti,ab,kw | **1013692** |
