# Supplementary material for: Descriptions of sham acupuncture in randomised controlled trials: a critical review of the literature
Source: BMC Complement Med Ther. 2023 May 30;23:173. doi: 10.1186/s12906-023-04007-7 (PMC10227975; doi:10.1186/s12906-023-04007-7)
Supplement: Supplementary file 3 — Supplementary Material 3 [file 12906_2023_4007_MOESM3_ESM.docx]

**Included trials:**

1. Gaw AC, Chang LW, Shaw LC. Efficacy of acupuncture on osteoarthritic pain. A controlled, double-blind study. N Engl J Med. 1975;293:375-8.
2. Moore ME, Berk SN. Acupuncture for chronic shoulder pain. An experimental study with attention to the role of placebo and hypnotic susceptibility. Ann Intern Med. 1976;84:381-4.
3. Edelist G, Gross AE, Langer F. Treatment of low back pain with acupuncture. Can Anaesth Soc J. 1976;23:303-6.
4. Berk SN, Moore ME, Resnick JH. Psychosocial factors as mediators of acupuncture therapy. J Consult Clin Psychol. 1977;45:612-9.
5. Knox VJ, Handfield-Jones CE, Shum K. Subject expectancy and the reduction of cold pressor pain with acupuncture and placebo acupuncture. Psychosom Med. 1979;41:477-86.
6. Lehmann TR, Russell DW, Spratt KF. The impact of patients with nonorganic physical findings on a controlled trial of transcutaneous electrical nerve stimulation and electroacupuncture. Spine (Phila Pa 1976). 1983;8:625-34.
7. Macdonald AJ, Macrae KD, Master BR, Rubin AP. Superficial acupuncture in the relief of chronic low back pain. Ann R Coll Surg Engl. 1983;65:44-6.
8. Dowson DI, Lewith GT, Machin D. The effects of acupuncture versus placebo in the treatment of headache. Pain. 1985;21:35-42.
9. Lehmann TR, Russell DW, Spratt KF, Colby H, Liu KY, Fairchild ML, et al. Efficacy of electroacupuncture and TENS in the rehabilitation of chronic low back pain patients. Pain. 1986;26:277-290.
10. Jobst K, Chen JH, McPherson K, Arrowsmith J, Brown V, Efthimiou J, et al. Controlled trial of acupuncture for disabling breathlessness. Lancet. 1986;2:1416-9.
11. Ballegaard S, Jensen G, Pedersen F, Nissen VH. Acupuncture in severe, stable angina pectoris: a randomized trial. Acta Med Scand. 1986;220:307-13.
12. Petrie JP, Hazleman BL. A controlled study of acupuncture in neck pain. Br J Rheumatol. 1986;25:271-5.
13. Bullock ML, Culliton PD, Olander RT. Controlled trial of acupuncture for severe recidivist alcoholism. Lancet. 1989;1:1435-9.
14. Vincent CA. A controlled trial of the treatment of migraine by acupuncture. Clin J Pain. 1989;5:305-12.
15. Ballegaard S, Pedersen F, Pietersen A, Nissen VH, Olsen NV. Effects of acupuncture in moderate, stable angina pectoris: a controlled study. J Intern Med. 1990;227:25-30.
16. Haker E, Lundeberg T. Laser treatment applied to acupuncture points in lateral humeral epicondylalgia. A double-blind study. Pain. 1990;43:243-247.
17. Tavola T, Gala C, Conte G, Invernizzi G. Traditional Chinese acupuncture in tension-type headache: a controlled study. Pain. 1992;48:325-329.
18. Worner TM, Zeller B, Schwarz H, Zwas F, Lyon D. Acupuncture fails to improve treatment outcome in alcoholics. Drug Alcohol Depend. 1992;30:169-73.
19. Molsberger A, Hille E. The analgesic effect of acupuncture in chronic tennis elbow pain. Br J Rheumatol. 1994;33:1162-5.
20. Hesse J, Møgelvang B, Simonsen H. Acupuncture versus metoprolol in migraine prophylaxis: a randomized trial of trigger point inactivation. J Intern Med. 1994;235:451-6.
21. Takeda W, Wessel J. Acupuncture for the treatment of pain of osteoarthritic knees. Arthritis Care Res. 1994;7:118-22.
22. Blom M, Dawidson I, Fernberg JO, Johnson G, Angmar-Månsson B. Acupuncture treatment of patients with radiation-induced xerostomia. Eur J Cancer B Oral Oncol. 1996;32B:182-90.
23. Schwager KL, Baines DB, Meyer RJ. Acupuncture and postoperative vomiting in day-stay paediatric patients. Anaesth Intensive Care. 1996;24:674-7.
24. Aune A, Alraek T, LiHua H, Baerheim A. Acupuncture in the prophylaxis of recurrent lower urinary tract infection in adult women. Scand J Prim Health Care. 1998;16:37-9.
25. al-Sadi M, Newman B, Julious SA. Acupuncture in the prevention of postoperative nausea and vomiting. Anaesthesia. 1997;52:658-61.
26. Wang B, Tang J, White PF, Naruse R, Sloninsky A, Kariger R, et al. Effect of the intensity of transcutaneous acupoint electrical stimulation on the postoperative analgesic requirement. Anesth Analg. 1997;85:406-13.
27. Pintov S, Lahat E, Alstein M, Vogel Z, Barg J. Acupuncture and the opioid system: implications in management of migraine. Pediatr Neurol. 1997;17:129-33.
28. NG Lavies. Laser acupuncture for migraine and muscle tension headache: a double-blind controlled trial. Acupuncture in medicine : journal of the British Medical Acupuncture Society. 1998; doi:10.1136/aim.16.2.73.
29. Biernacki W, Peake MD. Acupuncture in treatment of stable asthma. Respir Med. 1998;92:1143-5.
30. Schlager A, Offer T, Baldissera I. Laser stimulation of acupuncture point P6 reduces postoperative vomiting in children undergoing strabismus surgery. Br J Anaesth. 1998;81:529-32.
31. Vilholm OJ, Møller K, Jørgensen K. Effect of traditional Chinese acupuncture on severe tinnitus: a double-blind, placebo-controlled, clinical investigation with open therapeutic control. Br J Audiol. 1998;32:197-204.
32. Hamza MA, White PF, Ahmed HE, Ghoname EA. Effect of the frequency of transcutaneous electrical nerve stimulation on the postoperative opioid analgesic requirement and recovery profile. Anesthesiology. 1999;91:1232-8.
33. Kloster R, Larsson PG, Lossius R, Nakken KO, Dahl R, Xiu-Ling X, et al. The effect of acupuncture in chronic intractable epilepsy. Seizure. 1999;8:170-4.
34. Shen J, Wenger N, Glaspy J, Hays RD, Albert PS, Choi C, et al. Electroacupuncture for control of myeloablative chemotherapy-induced emesis: a randomized controlled trial. JAMA. 2000;284:2755-61.
35. Barlas P, Robinson J, Allen J, Baxter GD. Lack of effect of acupuncture upon signs and symptoms of delayed onset muscle soreness. Clin Physiol. 2000;20:449-56.
36. Röschke J, Wolf C, Müller MJ, Wagner P, Mann K, Grözinger M, et al. The benefit from whole body acupuncture in major depression. J Affect Disord. 2000;57:73-81.
37. Karst M, Rollnik JD, Fink M, Reinhard M, Piepenbrock S. Pressure pain threshold and needle acupuncture in chronic tension-type headache--a double-blind placebo-controlled study. Pain. 2000;88:199-203.
38. White AR, Resch KL, Chan JC, Norris CD, Modi SK, Patel JN, et al. Acupuncture for episodic tension-type headache: a multicentre randomized controlled trial. Cephalalgia. 2000;20:632-7.
39. Stavem K, Kloster R, Røssberg E, Larsson PG, Dahl R, Kinge E, et al. Acupuncture in intractable epilepsy: lack of effect on health-related quality of life. Seizure. 2000;9:422-6.
40. Knight B, Mudge C, Openshaw S, White A, Hart A. Effect of acupuncture on nausea of pregnancy: a randomized, controlled trial. Obstet Gynecol. 2001;97:184-8.
41. Irnich D, Behrens N, Molzen H, König A, Gleditsch J, Krauss M, et al. Randomised trial of acupuncture compared with conventional massage and "sham" laser acupuncture for treatment of chronic neck pain. BMJ. 2001;322:1574-8.
42. Karst M, Reinhard M, Thum P, Wiese B, Rollnik J, Fink M. Needle acupuncture in tension-type headache: a randomized, placebo-controlled study. Cephalalgia. 2001;21:637-42.
43. Habek D, Habek JC, Barbir A. Using acupuncture to treat premenstrual syndrome. Arch Gynecol Obstet. 2002;267:23-6.
44. Carlsson CP, Sjölund BH. Acupuncture for chronic low back pain: a randomized placebo-controlled study with long-term follow-up. Clin J Pain. 2001;17:296-305.
45. Molsberger AF, Mau J, Pawelec DB, Winkler J. Does acupuncture improve the orthopedic management of chronic low back pain--a randomized, blinded, controlled trial with 3 months follow up. Pain. 2002;99:579-587.
46. Sangdee C, Teekachunhatean S, Sananpanich K, Sugandhavesa N, Chiewchantanakit S, Pojchamarnwiputh S, et al. Electroacupuncture versus diclofenac in symptomatic treatment of osteoarthritis of the knee: a randomized controlled trial. BMC Complement Altern Med. 2002;2:3.
47. Sandberg M, Wijma K, Wyon Y, Nedstrand E, Hammar M. Effects of electro-acupuncture on psychological distress in postmenopausal women. Complement Ther Med. 2002;10:161-9.
48. Smith C, Crowther C. The placebo response and effect of time in a trial of acupuncture to treat nausea and vomiting in early pregnancy. Complement Ther Med. 2002;10:210-6.
49. Lin JG, Lo MW, Wen YR, Hsieh CL, Tsai SK, Sun WZ. The effect of high and low frequency electroacupuncture in pain after lower abdominal surgery. Pain. 2002;99:509-514.
50. Skilnand E, Fossen D, Heiberg E. Acupuncture in the management of pain in labor. Acta Obstet Gynecol Scand. 2002;81:943-8.
51. Nabeta T, Kawakita K. Relief of chronic neck and shoulder pain by manual acupuncture to tender points--a sham-controlled randomized trial. Complement Ther Med. 2002;10:217-22.
52. Rusy LM, Hoffman GM, Weisman SJ. Electroacupuncture prophylaxis of postoperative nausea and vomiting following pediatric tonsillectomy with or without adenoidectomy. Anesthesiology. 2002;96:300-5.
53. Sim CK, Xu PC, Pua HL, Zhang G, Lee TL. Effects of electroacupuncture on intraoperative and postoperative analgesic requirement. Acupunct Med. 2002;20:56-65.
54. Malmström M, Ahlner J, Carlsson C, Schmekel B. No effect of chinese acupuncture on isocapnic hyperventilation with cold air in asthmatics, measured with impulse oscillometry. Acupunct Med. 2002;20:66-73.
55. Näslund J, Näslund UB, Odenbring S, Lundeberg T. Sensory stimulation (acupuncture) for the treatment of idiopathic anterior knee pain. J Rehabil Med. 2002;34:231-8.
56. Shapira MY, Berkman N, Ben-David G, Avital A, Bardach E, Breuer R. Short-term acupuncture therapy is of no benefit in patients with moderate persistent asthma. Chest. 2002;121:1396-400.
57. Fink M, Wolkenstein E, Luennemann M, Gutenbrunner C, Gehrke A, Karst M. Chronic epicondylitis: effects of real and sham acupuncture treatment: a randomised controlled patient- and examiner-blinded long-term trial. Forsch Komplementarmed Klass Naturheilkd. 2002;9:210-5.
58. Medici TC, Grebski E, Wu J, Hinz G, Wüthrich B. Acupuncture and bronchial asthma: a long-term randomized study of the effects of real versus sham acupuncture compared to controls in patients with bronchial asthma. J Altern Complement Med. 2002;8:737-54.
59. Fink M, Wolkenstein E, Karst M, Gehrke A. Acupuncture in chronic epicondylitis: a randomized controlled trial. Rheumatology (Oxford). 2002;41:205-9.
60. Karst M, Passie T, Friedrich S, Wiese B, Schneider U. Acupuncture in the treatment of alcohol withdrawal symptoms: a randomized, placebo-controlled inpatient study. Addict Biol. 2002;7:415-9.
61. Leibing E, Leonhardt U, Köster G, Goerlitz A, Rosenfeldt JA, Hilgers R, et al. Acupuncture treatment of chronic low-back pain -- a randomized, blinded, placebo-controlled trial with 9-month follow-up. Pain. 2002;96:189-96.
62. Streitberger K, Friedrich-Rust M, Bardenheuer H, Unnebrink K, Windeler J, Goldschmidt H, et al. Effect of acupuncture compared with placebo-acupuncture at P6 as additional antiemetic prophylaxis in high-dose chemotherapy and autologous peripheral blood stem cell transplantation: a randomized controlled single-blind trial. Clin Cancer Res. 2003;9:2538-44.
63. Karst M, Scheinichen D, Rueckert T, Wagner T, Wiese B, Piepenbrock S, et al. Effect of acupuncture on the neutrophil respiratory burst: a placebo-controlled single-blinded study. Complement Ther Med. 2003;11:4-10.
64. Habek D, Cerkez Habek J, Bobić-Vuković M, Vujić B. Efficacy of acupuncture for the treatment of primary dysmenorrhea. Gynakol Geburtshilfliche Rundsch. 2003;43:250-3.
65. Irnich D, Karg H, Behrens N, et al. Controlled trial on point specificity of acupuncture in the treatment of lateral epicondylitis (Tennis Elbow). Physikalische Medizin Rehabilitationsmedizin Kurortmedizin. 2003;13:215-219.
66. Kvorning N, Christiansson C, Akeson J. Acupuncture facilitates neuromuscular and oculomotor responses to skin incision with no influence on auditory evoked potentials under sevoflurane anaesthesia. Acta Anaesthesiol Scand. 2003;47:1073-8.
67. Fanti L, Gemma M, Passaretti S, Guslandi M, Testoni PA, Casati A, et al. Electroacupuncture analgesia for colonoscopy. a prospective, randomized, placebo-controlled study. Am J Gastroenterol. 2003;98:312-6.
68. Kerr DP, Walsh DM, Baxter D. Acupuncture in the management of chronic low back pain: a blinded randomized controlled trial. Clin J Pain. 2003;19:364-70.
69. Hahn M, Steins A, Möhrle M, Blum A, Jünger M. Is there a vasospasmolytic effect of acupuncture in patients with secondary Raynaud phenomenon? J Dtsch Dermatol Ges. 2004;2:758-62.
70. Berman BM, Lao L, Langenberg P, Lee WL, Gilpin AM, Hochberg MC. Effectiveness of acupuncture as adjunctive therapy in osteoarthritis of the knee: a randomized, controlled trial. Ann Intern Med. 2004;141:901-10.
71. Yiu E, Xu JJ, Murry T, Wei WI, Yu M, Ma E, et al. A randomized treatment-placebo study of the effectiveness of acupuncture for benign vocal pathologies. J Voice. 2006;20:144-56.
72. Vas J, Méndez C, Perea-Milla E, Vega E, Panadero MD, León JM, et al. Acupuncture as a complementary therapy to the pharmacological treatment of osteoarthritis of the knee: randomised controlled trial. BMJ. 2004;329:1216.
73. Razavi M, Jansen GB. Effects of acupuncture and placebo TENS in addition to exercise in treatment of rotator cuff tendinitis. Clin Rehabil. 2004;18:872-8.
74. Kararmaz A, Kaya S, Karaman H, Turhanoglu S. Effect of the frequency of transcutaneous electrical nerve stimulation on analgesia during extracorporeal shock wave lithotripsy. Urol Res. 2004;32:411-5.
75. Guerra de Hoyos JA, Martín MDCA, Leon EBYB, Lopez MV, López TM, Morilla FAV, et al. Randomised trial of long term effect of acupuncture for shoulder pain. Pain. 2004;112:289-298.
76. Fink M, Rollnik JD, Bijak M, Borstädt C, Däuper J, Guergueltcheva V, et al. Needle acupuncture in chronic poststroke leg spasticity. Arch Phys Med Rehabil. 2004;85:667-72.
77. Magnusson AL, Svensson RE, Leirvik C, Gunnarsson RK. The effect of acupuncture on allergic rhinitis: a randomized controlled clinical trial. Am J Chin Med. 2004;32:105-15.
78. White P, Lewith G, Prescott P, Conway J. Acupuncture versus placebo for the treatment of chronic mechanical neck pain: a randomized, controlled trial. Ann Intern Med. 2004;141:911-9.
79. Joos S, Brinkhaus B, Maluche C, Maupai N, Kohnen R, Kraehmer N, et al. Acupuncture and moxibustion in the treatment of active Crohn's disease: a randomized controlled study. Digestion. 2004;69:131-9.
80. Streitberger K, Diefenbacher M, Bauer A, Conradi R, Bardenheuer H, Martin E, et al. Acupuncture compared to placebo-acupuncture for postoperative nausea and vomiting prophylaxis: a randomised placebo-controlled patient and observer blind trial. Anaesthesia. 2004;59:142-9.
81. Gan TJ, Jiao KR, Zenn M, Georgiade G. A randomized controlled comparison of electro-acupoint stimulation or ondansetron versus placebo for the prevention of postoperative nausea and vomiting. Anesth Analg. 2004;99:1070-1075.
82. Ng DK, Chow PY, Ming SP, Hong SH, Lau S, Tse D, et al. A double-blind, randomized, placebo-controlled trial of acupuncture for the treatment of childhood persistent allergic rhinitis. Pediatrics. 2004;114:1242-7.
83. Ilbuldu E, Cakmak A, Disci R, Aydin R. Comparison of laser, dry needling, and placebo laser treatments in myofascial pain syndrome. Photomed Laser Surg. 2004;22:306-11.
84. Linde M, Fjell A, Carlsson J, Dahlöf C. Role of the needling per se in acupuncture as prophylaxis for menstrually related migraine: a randomized placebo-controlled study. Cephalalgia. 2005;25:41-7.
85. Li Z, Wang C, Mak AF, Chow DH. Effects of acupuncture on heart rate variability in normal subjects under fatigue and non-fatigue state. Eur J Appl Physiol. 2005;94:633-40.
86. Rössberg E, Larsson PG, Birkeflet O, Söholt LE, Stavem K. Comparison of traditional Chinese acupuncture, minimal acupuncture at non-acupoints and conventional treatment for chronic sinusitis. Complement Ther Med. 2005;13:4-10.
87. Wayne PM, Krebs DE, Macklin EA, Schnyer R, Kaptchuk TJ, Parker SW, et al. Acupuncture for upper-extremity rehabilitation in chronic stroke: a randomized sham-controlled study. Arch Phys Med Rehabil. 2005;86:2248-55.
88. Quah-Smith JI, Tang WM, Russell J. Laser acupuncture for mild to moderate depression in a primary care setting--a randomised controlled trial. Acupunct Med. 2005;23:103-11.
89. Ebneshahidi NS, Heshmatipour M, Moghaddami A, Eghtesadi-Araghi P. The effects of laser acupuncture on chronic tension headache--a randomised controlled trial. Acupunct Med. 2005;23:13-8.
90. Park J, White AR, James MA, Hemsley AG, Johnson P, Chambers J, et al. Acupuncture for subacute stroke rehabilitation: a Sham-controlled, subject- and assessor-blind, randomized trial. Arch Intern Med. 2005;165:2026-31.
91. Forbes A, Jackson S, Walter C, Quraishi S, Jacyna M, Pitcher M. Acupuncture for irritable bowel syndrome: a blinded placebo-controlled trial. World J Gastroenterol. 2005;11:4040-4.
92. Emmons SL, Otto L. Acupuncture for overactive bladder: a randomized controlled trial. Obstet Gynecol. 2005;106:138-43.
93. Smith C, Coyle M, Norman RJ. Influence of acupuncture stimulation on pregnancy rates for women undergoing embryo transfer. Fertil Steril. 2006;85:1352-8.
94. Diener HC, Kronfeld K, Boewing G, Lungenhausen M, Maier C, Molsberger A, et al. Efficacy of acupuncture for the prophylaxis of migraine: a multicentre randomised controlled clinical trial. Lancet Neurol. 2006;5:310-6.
95. Manheimer E, Lim B, Lao L, Berman B. Acupuncture for knee osteoarthritis--a randomised trial using a novel sham. Acupunct Med. 2006;24 Suppl:S7-14.
96. Alecrim-Andrade J, Maciel-Júnior JA, Cladellas XC, Correa-Filho HR, Machado HC. Acupuncture in migraine prophylaxis: a randomized sham-controlled trial. Cephalalgia. 2006;26:520-9.
97. Habib AS, Itchon-Ramos N, Phillips-Bute BG, Gan TJ; Duke Women's Anesthesia (DWA) Research Group. Transcutaneous acupoint electrical stimulation with the ReliefBand for the prevention of nausea and vomiting during and after cesarean delivery under spinal anesthesia. Anesth Analg. 2006;102:581-4.
98. Vas J, Mendez C, Perea-Milla E. Acupuncture vs streitberger needle in knee osteoarthritis – an RCT. Acupuncture in Medicine. 2006; 24:15-24.
99. Vas J, Perea-Milla E, Méndez C, Sánchez Navarro C, León Rubio JM, et al. Efficacy and safety of acupuncture for chronic uncomplicated neck pain: a randomised controlled study. Pain. 2006;126:245-55.
100. Inoue M, Kitakoji H, Ishizaki N, Tawa M, Yano T, Katsumi Y, et al. Relief of low back pain immediately after acupuncture treatment--a randomised, placebo controlled trial. Acupunct Med. 2006;24:103-8.
101. Gioia L, Cabrini L, Gemma M, Fiori R, Fasce F, Bolognesi G, et al. Sedative effect of acupuncture during cataract surgery: prospective randomized double-blind study. J Cataract Refract Surg. 2006;32:1951-4.
102. Joos S, Wildau N, Kohnen R, Szecsenyi J, Schuppan D, Willich SN, et al. Acupuncture and moxibustion in the treatment of ulcerative colitis: a randomized controlled study. Scand J Gastroenterol. 2006;41:1056-63.
103. Aigner N, Fialka C, Radda C, Vecsei V. Adjuvant laser acupuncture in the treatment of whiplash injuries: a prospective, randomized placebo-controlled trial. Wien Klin Wochenschr. 2006;118:95-9.
104. Schneider A, Enck P, Streitberger K, Weiland C, Bagheri S, Witte S, et al. Acupuncture treatment in irritable bowel syndrome. Gut. 2006;55:649-54.
105. Cabrini L, Gioia L, Gemma M, Melloni G, Carretta A, Ciriaco P, et al. Acupuncture for diagnostic fiberoptic bronchoscopy: a prospective, randomized, placebo-controlled study. Am J Chin Med. 2006;34:409-15.
106. Haake M, Müller HH, Schade-Brittinger C, Basler HD, Schäfer H, Maier C, et al. German Acupuncture Trials (GERAC) for chronic low back pain: randomized, multicenter, blinded, parallel-group trial with 3 groups. Arch Intern Med. 2007;16:1892-8.
107. Yurtkuran M, Alp A, Konur S, Ozçakir S, Bingol U. Laser acupuncture in knee osteoarthritis: a double-blind, randomized controlled study. Photomed Laser Surg. 2007;25:14-20.
108. Tsang RC, Tsang PL, Ko CY, Kong BC, Lee WY, Yip HT. Effects of acupuncture and sham acupuncture in addition to physiotherapy in patients undergoing bilateral total knee arthroplasty--a randomized controlled trial. Clin Rehabil. 2007;21:719-28.
109. Song Y, Zhou D, Fan J, Luo H, Halbreich U. Effects of electroacupuncture and fluoxetine on the density of GTP-binding-proteins in platelet membrane in patients with major depressive disorder. J Affect Disord. 2007;98:253-7.
110. Endres HG, Böwing G, Diener HC, Lange S, Maier C, Molsberger A, et al. Acupuncture for tension-type headache: a multicentre, sham-controlled, patient-and observer-blinded, randomised trial. J Headache Pain. 2007;8:306-14.
111. Shen YF, Goddard G. The short-term effects of acupuncture on myofascial pain patients after clenching. Pain Pract. 2007;7:256-64.
112. Smith P, Mosscrop D, Davies S, Sloan P, Al-Ani Z. The efficacy of acupuncture in the treatment of temporomandibular joint myofascial pain: a randomised controlled trial. J Dent. 2007;35:259-67.
113. Chao AS, Chao A, Wang TH, Chang YC, Peng HH, Chang SD, et al. Pain relief by applying transcutaneous electrical nerve stimulation (TENS) on acupuncture points during the first stage of labor: a randomized double-blind placebo-controlled trial. Pain. 2007;127:214-220.
114. Flachskampf FA, Gallasch J, Gefeller O, Gan J, Mao J, Pfahlberg AB, et al. Randomized trial of acupuncture to lower blood pressure. Circulation. 2007;115:3121-9.
115. Dyson-Hudson TA, Kadar P, LaFountaine M, Emmons R, Kirshblum SC, Tulsky D, et al. Acupuncture for chronic shoulder pain in persons with spinal cord injury: a small-scale clinical trial. Arch Phys Med Rehabil. 2007;88:1276-83.
116. Yin C, Seo B, Park HJ, Cho M, Jung W, Choue R, et al. Acupuncture, a promising adjunctive therapy for essential hypertension: a double-blind, randomized, controlled trial. Neurol Res. 2007;29:S98-103.
117. Xue CC, An X, Cheung TP, Da Costa C, Lenon GB, Thien FC, et al. Acupuncture for persistent allergic rhinitis: a randomised, sham-controlled trial. Med J Aust. 2007;187:337-41.
118. Nir Y, Huang MI, Schnyer R, Chen B, Manber R. Acupuncture for postmenopausal hot flashes. Maturitas. 2007;56:383-95.
119. Schaechter JD, Connell BD, Stason WB, Kaptchuk TJ, Krebs DE, Macklin EA, et al. Correlated change in upper limb function and motor cortex activation after verum and sham acupuncture in patients with chronic stroke. J Altern Complement Med. 2007;13:527-32.
120. Hübscher M, Vogt L, Bernhörster M, Rosenhagen A, Banzer W. Effects of acupuncture on symptoms and muscle function in delayed-onset muscle soreness. J Altern Complement Med. 2008;14:1011-6.
121. Jubb RW, Tukmachi ES, Jones PW, Dempsey E, Waterhouse L, Brailsford S. A blinded randomised trial of acupuncture (manual and electroacupuncture) compared with a non-penetrating sham for the symptoms of osteoarthritis of the knee. Acupunct Med. 2008;26:69-78.
122. Gottschling S, Meyer S, Gribova I, Distler L, Berrang J, Gortner L, et al. Laser acupuncture in children with headache: a double-blind, randomized, bicenter, placebo-controlled trial. Pain. 2008;137:405-412.
123. Hopwood V, Lewith G, Prescott P, Campbell MJ. Evaluating the efficacy of acupuncture in defined aspects of stroke recovery: a randomised, placebo controlled single blind study. J Neurol. 2008;255:858-66.
124. DeSantana JM, Santana-Filho VJ, Guerra DR, Sluka KA, Gurgel RQ, da Silva WM Jr. Hypoalgesic effect of the transcutaneous electrical nerve stimulation following inguinal herniorrhaphy: a randomized, controlled trial. J Pain. 2008;9:623-9.
125. Vas J, Ortega C, Olmo V, Perez-Fernandez F, Hernandez L, Medina I, et al. Single-point acupuncture and physiotherapy for the treatment of painful shoulder: a multicentre randomized controlled trial. Rheumatology (Oxford). 2008;47:887-93.
126. Li L, Liu H, Li YZ, Xu JY, Shan BC, Gong D, et al. The human brain response to acupuncture on same-meridian acupoints: evidence from an fMRI study. J Altern Complement Med. 2008;14:673-8.
127. Cho JH, Chung WK, Kang W, Choi SM, Cho CK, Son CG. Manual acupuncture improved quality of life in cancer patients with radiation-induced xerostomia. J Altern Complement Med. 2008;14:523-6.
128. Facco E, Liguori A, Petti F, Zanette G, Coluzzi F, De Nardin M, et al. Traditional acupuncture in migraine: a controlled, randomized study. Headache. 2008;48:398-407.
129. Alecrim-Andrade J, Maciel-Júnior JA, Carnè X, Severino Vasconcelos GM, Correa-Filho HR. Acupuncture in migraine prevention: a randomized sham controlled study with 6-months posttreatment follow-up. Clin J Pain. 2008;24:98-105.
130. Goldman RH, Stason WB, Park SK, Kim R, Schnyer RN, Davis RB, et al. Acupuncture for treatment of persistent arm pain due to repetitive use: a randomized controlled clinical trial. Clin J Pain. 2008;24:211-8.
131. Lee SW, Liong ML, Yuen KH, Leong WS, Chee C, Cheah PY, et al. Acupuncture versus sham acupuncture for chronic prostatitis/chronic pelvic pain. Am J Med. 2008;121:79.e1-7.
132. Lau KS, Jones AY. A single session of Acu-TENS increases FEV1 and reduces dyspnoea in patients with chronic obstructive pulmonary disease: a randomised, placebo-controlled trial. Aust J Physiother. 2008;54:179-84.
133. Bäcker M, Grossman P, Schneider J, Michalsen A, Knoblauch N, Tan L, et al. Acupuncture in migraine: investigation of autonomic effects. Clin J Pain. 2008;24:106-15.
134. Elden H, Fagevik-Olsen M, Ostgaard HC, Stener-Victorin E, Hagberg H. Acupuncture as an adjunct to standard treatment for pelvic girdle pain in pregnant women: randomised double-blinded controlled trial comparing acupuncture with non-penetrating sham acupuncture. BJOG. 2008;115:1655-68.
135. Haiko Sprott MD. Efficiency of acupuncture in patients with fibromyalgia. Clinical Bulletin of Myofascial Therapy. 1998; doi:[10.1300/J425v03n01_05](https://doi.org/10.1300/J425v03n01_05).
136. Zheng LH, Sun H, Wang GN, Liang J, Wu HX. Effect of transcutaneous electrical acupoint stimulation on nausea and vomiting induced by patient controlled intravenous analgesia with tramadol. Chin J Integr Med. 2008;14:61-4.
137. Liu YY, Duan SE, Cai MX, Zou P, Lai Y, Li YL. Evaluation of transcutaneous electroacupoint stimulation with the train-of-four mode for preventing nausea and vomiting after laparoscopic cholecystectomy. Chin J Integr Med. 2008;14:94-7.
138. Jing C, Gaohua W, Ling X, Huiling W, Xiaoping W, Chengyan L. Electro-acupuncture versus sham electro-acupuncture for auditory hallucinations in patients with schizophrenia: a randomized controlled trial. Clin Rehabil. 2009;23:579-88.
139. Lathia AT, Jung SM, Chen LX. Efficacy of acupuncture as a treatment for chronic shoulder pain. J Altern Complement Med. 2009;15:613-8.
140. Lin ZP, Lan LW, He TY, Lin SP, Lin JG, Jang TR, et al. Effects of acupuncture stimulation on recovery ability of male elite basketball athletes. Am J Chin Med. 2009;37:471-81.
141. Yeung WF, Chung KF, Zhang SP, Yap TG, Law AC. Electroacupuncture for primary insomnia: a randomized controlled trial. Sleep. 2009;32:1039-47.
142. Lee SH, Lee BC. Electroacupuncture relieves pain in men with chronic prostatitis/chronic pelvic pain syndrome: three-arm randomized trial. Urology. 2009;73:1036-41.
143. Kong J, Kaptchuk TJ, Polich G, Kirsch I, Vangel M, Zyloney C, et al. Expectancy and treatment interactions: a dissociation between acupuncture analgesia and expectancy evoked placebo analgesia. Neuroimage. 2009;45:940-9.
144. Anzinger A, Albrecht J, Kopietz R, Kleemann AM, Schöpf V, Demmel M, et al. Effects of laserneedle acupuncture on olfactory sensitivity of healthy human subjects: a placebo-controlled, double-blinded, randomized trial. Rhinology. 2009;47:153-9.
145. Chang S, Chang ZG, Li SJ, Chiang MJ, Ma CM, Cheng HY, et al. Effects of acupuncture at Neiguan (PC 6) on electroencephalogram. Chin J Physiol. 2009;52:1-7.
146. Song C, Halbreich U, Han C, Leonard BE, Luo H. Imbalance between pro- and anti-inflammatory cytokines, and between Th1 and Th2 cytokines in depressed patients: the effect of electroacupuncture or fluoxetine treatment. Pharmacopsychiatry. 2009;42:182-8.
147. Yao S, Yao X. W2071 Effect of acupuncture on proximal gastric motility in patients with functional dyspepsia: a randomized, controlled clinical study. Gastroenterology. 2009;136:A-785.
148. Chou LW, Hsieh YL, Kao MJ, Hong CZ. Remote influences of acupuncture on the pain intensity and the amplitude changes of endplate noise in the myofascial trigger point of the upper trapezius muscle. Arch Phys Med Rehabil. 2009;90:905-12.
149. Yan T, Hui-Chan CW. Transcutaneous electrical stimulation on acupuncture points improves muscle function in subjects after acute stroke: a randomized controlled trial. J Rehabil Med. 2009;41:312-6.
150. Fang J, Jin Z, Wang Y, Li K, Kong J, Nixon EE, et al. The salient characteristics of the central effects of acupuncture needling: limbic-paralimbic-neocortical network modulation. Hum Brain Mapp. 2009;30:1196-206.
151. Wu JH, Chen HY, Chang YJ, Wu HC, Chang WD, Chu YJ, et al. Study of autonomic nervous activity of night shift workers treated with laser acupuncture. Photomed Laser Surg. 2009;27:273-9.
152. Chae Y, Lee H, Kim H, Sohn H, Park JH, Park HJ. The neural substrates of verum acupuncture compared to non-penetrating placebo needle: an fMRI study. Neurosci Lett. 2009;450:80-4.
153. Chae Y, Lee H, Kim H, Kim CH, Chang DI, Kim KM, et al. Parsing brain activity associated with acupuncture treatment in Parkinson's diseases. Mov Disord. 2009;24:1794-802.
154. Harris RE, Zubieta JK, Scott DJ, Napadow V, Gracely RH, Clauw DJ. Traditional Chinese acupuncture and placebo (sham) acupuncture are differentiated by their effects on mu-opioid receptors (MORs). Neuroimage. 2009;47:1077-85.
155. Fleckenstein J, Lill C, Lüdtke R, Gleditsch J, Rasp G, Irnich D. A single point acupuncture treatment at large intestine meridian: a randomized controlled trial in acute tonsillitis and pharyngitis. Clin J Pain. 2009;25:624-31.
156. Cherkin DC, Sherman KJ, Avins AL, Erro JH, Ichikawa L, Barlow WE, et al. A randomized trial comparing acupuncture, simulated acupuncture, and usual care for chronic low back pain. Arch Intern Med. 2009;169:858-66.
157. Zhang WJ, Yang XB, Zhong BL. Combination of acupuncture and fluoxetine for depression: a randomized, double-blind, sham-controlled trial. J Altern Complement Med. 2009;15:837-44.
158. Asher GN, Coeytaux RR, Chen W, Reilly AC, Loh YL, Harper TC. Acupuncture to initiate labor (Acumoms 2): a randomized, sham-controlled clinical trial. J Matern Fetal Neonatal Med. 2009;22:843-8.
159. Kim MS, Cho YC, Moon JH, Pak SC. A characteristic estimation of bio-signals for electro-acupuncture stimulations in human subjects. Am J Chin Med. 2009;37:505-17.
160. Yeh ML, Chang CY, Chu NF, Chen HH. A six-week acupoint stimulation intervention for quitting smoking. Am J Chin Med. 2009;37:829-36.
161. Li Y, Liang F, Yang X, Tian X, Yan J, Sun G, et al. Acupuncture for treating acute attacks of migraine: a randomized controlled trial. Headache. 2009;49:805-16.
162. Hervik J, Mjåland O. Acupuncture for the treatment of hot flashes in breast cancer patients, a randomized, controlled trial. Breast Cancer Res Treat. 2009;116:311-6.
163. So EW, Ng EH, Wong YY, Lau EY, Yeung WS, Ho PC. A randomized double blind comparison of real and placebo acupuncture in IVF treatment. Hum Reprod. 2009;24:341-8.
164. Dieterle S, Li C, Greb R, Bartzsch F, Hatzmann W, Huang D. A prospective randomized placebo-controlled study of the effect of acupuncture in infertile patients with severe oligoasthenozoospermia. Fertil Steril. 2009;92:1340-1343.
165. Lembo AJ, Conboy L, Kelley JM, Schnyer RS, McManus CA, Quilty MT, et al. A treatment trial of acupuncture in IBS patients. Am J Gastroenterol. 2009;104:1489-97.
166. Tong Y, Jia Q, Sun Y, Hou Z, Wang Y. Acupuncture in the treatment of diabetic bladder dysfunction. J Altern Complement Med. 2009 ;15:905-9.
167. Fu WB, Liang ZH, Zhu XP, Yu P, Zhang JF. Analysis on the effect of acupuncture in treating cervical spondylosis with different syndrome types. Chin J Integr Med. 2009;15:426-30.
168. Kong J, Kaptchuk TJ, Polich G, Kirsch I, Vangel M, Zyloney C, et al. An fMRI study on the interaction and dissociation between expectation of pain relief and acupuncture treatment. Neuroimage. 2009;47:1066-76.
169. Fu WB, Fan L, Zhu XP, He Q, Wang L, Zhuang LX, et al. Depressive neurosis treated by acupuncture for regulating the liver--a report of 176 cases. J Tradit Chin Med. 2009;29:83-6.
170. Ahn AC, Schnyer R, Conboy L, Laufer MR, Wayne PM. Electrodermal measures of Jing-Well points and their clinical relevance in endometriosis-related chronic pelvic pain. J Altern Complement Med. 2009;15:1293-305.
171. Sahmeddini MA, Farbood A, Ghafaripuor S. Electro-acupuncture for pain relief after nasal septoplasty: a randomized controlled study. J Altern Complement Med. 2010;16:53-7.
172. Lu TW, Wei IP, Liu YH, Hsu WC, Wang TM, Chang CF, et al. Immediate effects of acupuncture on gait patterns in patients with knee osteoarthritis. Chin Med J (Engl). 2010;123:165-72.
173. Wu Y, Jin Z, Li K, Lu ZL, Wong V, Han TL, et al. Functional magnetic resonance imaging activation of the brain in children: real acupoint versus sham acupoint. J Child Neurol. 2010;25:849-55.
174. O'Brien KA, Varigos E, Black C, Komesaroff PA. Laser acupuncture does not improve menopausal symptoms. Menopause. 2010;17:636-41.
175. Zyloney CE, Jensen K, Polich G, Loiotile RE, Cheetham A, LaViolette PS, et al. Imaging the functional connectivity of the Periaqueductal Gray during genuine and sham electroacupuncture treatment. Mol Pain. 2010;6:80.
176. Ngai SP, Jones AY, Hui-Chan CW, Ko FW, Hui DS. Effect of 4 weeks of Acu-TENS on functional capacity and beta-endorphin level in subjects with chronic obstructive pulmonary disease: a randomized controlled trial. Respir Physiol Neurobiol. 2010;173:29-36.
177. Tong Y, Guo H, Han B. Fifteen-day acupuncture treatment relieves diabetic peripheral neuropathy. J Acupunct Meridian Stud. 2010;3:95-103.
178. Dias M, Carneiro NM, Guerra LA, Velarde GC, de Souza PA, da Silva LL, et al. Effects of electroacupuncture on local anaesthesia for inguinal hernia repair: a randomised placebo-controlled trial. Acupunct Med. 2010;28:65-70.
179. Li S, Yu B, Lin Z, Jiang S, He J, Kang L, et al. Randomized-controlled study of treating attention deficit hyperactivity disorder of preschool children with combined electro-acupuncture and behavior therapy. Complement Ther Med. 2010;18:175-83.
180. Sun MY, Hsieh CL, Cheng YY, Hung HC, Li TC, Yen SM, et al. The therapeutic effects of acupuncture on patients with chronic neck myofascial pain syndrome: a single-blind randomized controlled trial. Am J Chin Med. 2010;38:849-59.
181. Kokkotou E, Conboy LA, Ziogas DC, Quilty MT, Kelley JM, Davis RB, et al. Serum correlates of the placebo effect in irritable bowel syndrome. Neurogastroenterol Motil. 2010;22:285-e81.
182. Wong VC, Sun JG. Randomized controlled trial of acupuncture versus sham acupuncture in autism spectrum disorder. J Altern Complement Med. 2010;16:545-53.
183. Suarez-Almazor ME, Looney C, Liu Y, Cox V, Pietz K, Marcus DM, et al. A randomized controlled trial of acupuncture for osteoarthritis of the knee: effects of patient-provider communication. Arthritis Care Res (Hoboken). 2010;62:1229-36.
184. Modlock J, Nielsen BB, Uldbjerg N. Acupuncture for the induction of labour: a double-blind randomised controlled study. BJOG. 2010;117:1255-61.
185. Beer TM, Benavides M, Emmons SL, Hayes M, Liu G, Garzotto M, et al. Acupuncture for hot flashes in patients with prostate cancer. Urology. 2010;76:1182-8.
186. Venzke L, Calvert JF Jr, Gilbertson B. A randomized trial of acupuncture for vasomotor symptoms in post-menopausal women. Complement Ther Med. 2010;18:59-66.
187. Wang K, Bugge J, Bugge S. A randomised, placebo-controlled trial of manual and electrical acupuncture for the treatment of tinnitus. Complement Ther Med. 2010;18:249-55.
188. Hyun MK, Lee MS, Kang K, Choi SM. Body Acupuncture for Nicotine Withdrawal Symptoms: a Randomized Placebo-controlled Trial. Evid Based Complement Alternat Med. 2010;7:233-8.
189. So EW, Ng EH, Wong YY, Yeung WS, Ho PC. Acupuncture for frozen-thawed embryo transfer cycles: a double-blind randomized controlled trial. Reprod Biomed Online. 2010;20:814-21.
190. Yeh ML, Chung YC, Chen KM, Tsou MY, Chen HH. Acupoint electrical stimulation reduces acute postoperative pain in surgical patients with patient-controlled analgesia: a randomized controlled study. Altern Ther Health Med. 2010;16:10-8.
191. Jiang YH, Jiang W, Jiang LM, Lin GX, Yang H, Tan Y, et al. Clinical efficacy of acupuncture on the morphine-related side effects in patients undergoing spinal-epidural anesthesia and analgesia. Chin J Integr Med. 2010;16:71-4.
192. Boutouyrie P, Corvisier R, Ong K T, et al. Acute and chronic effects of acupuncture on radial artery: a randomized double blind study in migraine. Artery Research. 2010;4(1):7-14.
193. Cheung, M, Chan, et al. P24-4 Change in EEG activity associated with positive mood change after cutaneous stimulation over Shenmen. Clinical Neurophysiology. 2010;121:S245.
194. Leung WW, Ng SS, Jones AY, et al. A randomized study to evaluate the efficacy and mechanism of electroacupuncture in reducing discomfort associated with barostat-induced rectal distension. Gastrointestinal Endoscopy. 2010; 71:AB254-AB254.
195. Shin MS, Kim JI, Lee MS, Kim KH, Choi JY, Kang KW, et al. Acupuncture for treating dry eye: a randomized placebo-controlled trial. Acta Ophthalmol. 2010;88:e328-33.
196. Andersen D, Løssl K, Nyboe Andersen A, Fürbringer J, Bach H, Simonsen J, et al. Acupuncture on the day of embryo transfer: a randomized controlled trial of 635 patients. Reprod Biomed Online. 2010;21:366-72.
197. Liu CZ, Xie JP, Wang LP, Zheng YY, Ma ZB, Yang H, et al. Immediate analgesia effect of single point acupuncture in primary dysmenorrhea: a randomized controlled trial. Pain Med. 2011;12:300-7.
198. Yeung WF, Chung KF, Tso KC, Zhang SP, Zhang ZJ, Ho LM. Electroacupuncture for residual insomnia associated with major depressive disorder: a randomized controlled trial. Sleep. 2011;34:807-15.
199. Leung WW, Jones AY, Ng SS, Wong CY, Lee JF. Electroacupuncture in reduction of discomfort associated with barostat-induced rectal distension--a randomized controlled study. J Gastrointest Surg. 2011;15:660-6.
200. Liu J, Qin W, Guo Q, Sun J, Yuan K, Dong M, et al. Divergent neural processes specific to the acute and sustained phases of verum and SHAM acupuncture. J Magn Reson Imaging. 2011;33:33-40.
201. Ma W, Bai W, Lin C, Zhou P, Xia L, Zhao C, et al. Effects of Sanyinjiao (SP6) with electroacupuncture on labour pain in women during labour. Complement Ther Med. 2011;19 Suppl 1:S13-8.
202. Zhang R, Feng XJ, Guan Q, Cui W, Zheng Y, Sun W, et al. Increase of success rate for women undergoing embryo transfer by transcutaneous electrical acupoint stimulation: a prospective randomized placebo-controlled study. Fertil Steril. 2011;96:912-6.
203. Karaman MI, Koca O, Küçük EV, Öztürk M, Güneş M, Kaya C. Laser acupuncture therapy for primary monosymptomatic nocturnal enuresis. J Urol. 2011;185:1852-6.
204. Lomuscio A, Belletti S, Battezzati PM, Lombardi F. Efficacy of acupuncture in preventing atrial fibrillation recurrences after electrical cardioversion. J Cardiovasc Electrophysiol. 2011;22:241-7.
205. El-Deeb AM, Ahmady MS. Effect of acupuncture on nausea and/or vomiting during and after cesarean section in comparison with ondansetron. J Anesth. 2011;25:698-703.
206. Radvanska E, Kamperis K, Kleif A, Kovács L, Rittig S. Effect of laser acupuncture for monosymptomatic nocturnal enuresis on bladder reservoir function and nocturnal urine output. J Urol. 2011;185:1857-61.
207. Gemmell H, Hilland A. Immediate effect of electric point stimulation (TENS) in treating latent upper trapezius trigger points: a double blind randomised placebo-controlled trial. J Bodyw Mov Ther. 2011;15:348-54.
208. Yu JS, Shen KH, Chen WC, Her JS, Hsieh CL. Effects of electroacupuncture on benign prostate hyperplasia patients with lower urinary tract symptoms: a single-blinded, randomized controlled trial. Evid Based Complement Alternat Med. 2011;2011:303198.
209. Penza P, Bricchi M, Scola A, Campanella A, Lauria G. Electroacupuncture is not effective in chronic painful neuropathies. Pain Med. 2011;12:1819-23.
210. Liu J, Ng E. Does acupuncture improve pregnancy rate in sub-fertile women undergoing frozen–thawed embryo transfer cycles. Focus on Alternative & Complementary Therapies. 2011;16:137-138.
211. Kim YH, Kim KS, Lee HJ, Shim JC, Yoon SW. The efficacy of several neuromuscular monitoring modes at the P6 acupuncture point in preventing postoperative nausea and vomiting. Anesth Analg. 2011;112:819-23.
212. Chou LW, Hsieh YL, Chen HS, Hong CZ, Kao MJ, Han TI. Remote therapeutic effectiveness of acupuncture in treating myofascial trigger point of the upper trapezius muscle. Am J Phys Med Rehabil. 2011;90:1036-49.
213. Pastore LM, Williams CD, Jenkins J, Patrie JT. True and sham acupuncture produced similar frequency of ovulation and improved LH to FSH ratios in women with polycystic ovary syndrome. J Clin Endocrinol Metab. 2011;96:3143-50.
214. Ng MC, Jones AY, Cheng LC. The role of acu-TENS in hemodynamic recovery after open-heart surgery. Evid Based Complement Alternat Med. 2011;2011:301974.
215. Yeh ML, Chung YC, Chen KM, Chen HH. Pain reduction of acupoint electrical stimulation for patients with spinal surgery: a placebo-controlled study. Int J Nurs Stud. 2011;48:703-9.
216. Man KM, Man SS, Shen JL, Law KS, Chen SL, Liaw WJ, et al. Transcutaneous electrical nerve stimulation on ST36 and SP6 acupoints prevents hyperglycaemic response during anaesthesia: a randomised controlled trial. Eur J Anaesthesiol. 2011;28:420-6.
217. Sunay D, Ozdiken M, Arslan H, Seven A, Aral Y. The effect of acupuncture on postmenopausal symptoms and reproductive hormones: a sham controlled clinical trial. Acupunct Med. 2011;29:27-31.
218. Darbandi S, Darbandi M, Mobarhan M G, et al. The effects of electro acupuncture on leptin hormone in Iranian obese and overweight subjects. Clinical Biochemistry. 2011; doi:10.1016/j.clinbiochem.2011.08.302.
219. Taghavi R, Mogharabian N, Ataeian S J, et al. The effect of acupuncture on relieving pain following inguinal surgeries. Urology. 2011; doi:10.1016/j.urology.2011.07.1104.
220. Mackenzie IZ, Xu J, Cusick C, Midwinter-Morten H, Meacher H, Mollison J, et al. Acupuncture for pain relief during induced labour in nulliparae: a randomised controlled study. BJOG. 2011;118:440-7.
221. Cameron ID, Wang E, Sindhusake D. A randomized trial comparing acupuncture and simulated acupuncture for subacute and chronic whiplash. Spine (Phila Pa 1976). 2011;36:E1659-65.
222. Smith CA, Crowther CA, Petrucco O, Beilby J, Dent H. Acupuncture to treat primary dysmenorrhea in women: a randomized controlled trial. Evid Based Complement Alternat Med. 2011;2011:612464.
223. Chae Y, Um SI, Yi SH, Lee H, Chang DS, Yin CS, et al. Comparison of biomechanical properties between acupuncture and non-penetrating sham needle. Complement Ther Med. 2011;19 Suppl 1:S8-S12.
224. Lambert C, Berlin I, Lee TL, Hee SW, Tan AS, Picard D, et al. A standardized transcutaneous electric acupoint stimulation for relieving tobacco urges in dependent smokers. Evid Based Complement Alternat Med. 2011;2011:195714.
225. Sunay D, Sunay M, Aydoğmuş Y, Bağbancı S, Arslan H, Karabulut A, et al. Acupuncture versus paroxetine for the treatment of premature ejaculation: a randomized, placebo-controlled clinical trial. Eur Urol. 2011;59:765-71.
226. Francis RP, Marchant P, Johnson MI. Conventional versus acupuncture-like transcutaneous electrical nerve stimulation on cold-induced pain in healthy human participants: effects during stimulation. Clin Physiol Funct Imaging. 2011;31:363-70.
227. Chang BH, Sommers E. Acupuncture and the relaxation response for treating gastrointestinal symptoms in HIV patients on highly active antiretroviral therapy. Acupunct Med. 2011;29:180-7.
228. de Luca AC, da Fonseca AM, Lopes CM, Bagnoli VR, Soares JM, Baracat EC. Acupuncture-ameliorated menopausal symptoms: single-blind, placebo-controlled, randomized trial. Climacteric. 2011;14:140-5.
229. Miller E, Maimon Y, Rosenblatt Y, Mendler A, Hasner A, Barad A, et al. Delayed effect of acupuncture treatment in OA of the knee: A blinded, randomized, controlled trial. Evid Based Complement Alternat Med. 2011;2011:792975.
230. Chen MJ, Thompson T, Kropotov J, Gruzelier JH. Beneficial effects of electrostimulation contingencies on sustained attention and electrocortical activity. CNS Neurosci Ther. 2011;17:311-26.
231. Chae Y, Park HJ, Kang OS, Lee HJ, Kim SY, Yin CS, et al. Acupuncture attenuates autonomic responses to smoking-related visual cues. Complement Ther Med. 2011;19 Suppl 1:S1-7.
232. Jones AY, Ngai SP, Hui-Chan CW, Yu HP. Acute effects of acu-TENS on FEV1 and blood Β-endorphin level in chronic obstructive pulmonary disease. Altern Ther Health Med. 2011;17:8-13.
233. Sunay D, Sunay M, Aydoğmuş Y, Bağbancı S, Arslan H, Karabulut A, et al. Acupuncture versus paroxetine for the treatment of premature ejaculation: a randomized, placebo-controlled clinical trial. Eur Urol. 2011;59:765-71.
234. Lau, RKW, Lau, TFO, Cheng, CPY, Law, SW, et al. A double-blinded and randomized controlled trial for the effectiveness of electro-acupuncture for the management of signs and symptoms associated with collapse of spine. Hong Kong Physiotherapy Journal. 2011; doi:10.1016/j.hkpj.2011.08.025.
235. De Carvalho AO, Cabral L, Rubini E. Acupuncture improves flexibility: acute effect of acupuncture before a static stretch of hip adductors. Medical Acupuncture. 2011;23:27-33.
236. Amand M, Nguyen-Huu F, Balestra C. Acupuncture effect on thermal tolerance and electrical pain threshold: a randomised controlled trial. Acupunct Med. 2011;29:47-50.
237. Kim DI, Jeong JC, Kim KH, Rho JJ, Choi MS, Yoon SH, et al. Acupuncture for hot flushes in perimenopausal and postmenopausal women: a randomised, sham-controlled trial. Acupunct Med. 2011;29:249-56.
238. Deneen K, Wei Q, Peng L, et al. Randomized fMRI trial of the central effects of acute acupuncture on glucose levels and core body temperature in "overweight" males. Medical Acupuncture. 2011;23:165-173.
239. Wang LP, Zhang XZ, Guo J, Liu HL, Zhang Y, Liu CZ, et al. Efficacy of acupuncture for migraine prophylaxis: a single-blinded, double-dummy, randomized controlled trial. Pain. 2011;152:1864-1871.
240. Yeh BY, Hsu YC, Huang JY, Shih IT, Zhuo WJ, Tsai YF, et al. Effect of electroacupuncture in postanesthetic shivering during regional anesthesia: a randomized controlled trial. BMC Complement Altern Med. 2012;12:233.
241. Wang LP, Zhang XZ, Guo J, Liu HL, Zhang Y, Liu CZ, et al. Efficacy of acupuncture for acute migraine attack: a multicenter single blinded, randomized controlled trial. Pain Med. 2012;13:623-30.
242. Franasiak J, Young SL, Williams CD, Pastore LM. Longitudinal anti-müllerian hormone in women with polycystic ovary syndrome: an acupuncture randomized clinical trial. Evid Based Complement Alternat Med. 2012;2012:973712.
243. Chu WC, Wu JC, Yew DT, Zhang L, Shi L, Yeung DK, et al. Does acupuncture therapy alter activation of neural pathway for pain perception in irritable bowel syndrome?: a comparative study of true and sham acupuncture using functional magnetic resonance imaging. J Neurogastroenterol Motil. 2012;18:305-16.
244. Liu H, Xu J, Shan B, Li Y, Li L, Xue J, et al. Determining the precise cerebral response to acupuncture: an improved FMRI study. PLoS One. 2012;7:e49154.
245. Quispe-Cabanillas JG, Damasceno A, von Glehn F, Brandão CO, Damasceno BP, Silveira WD, et al. Impact of electroacupuncture on quality of life for patients with Relapsing-Remitting Multiple Sclerosis under treatment with immunomodulators: a randomized study. BMC Complement Altern Med. 2012;12:209.
246. Lin ML, Wu HC, Hsieh YH, Su CT, Shih YS, Lin CW, et al. Evaluation of the effect of laser acupuncture and cupping with ryodoraku and visual analog scale on low back pain. Evid Based Complement Alternat Med. 2012;2012:521612.
247. Zeng F, Qin W, Ma T, Sun J, Tang Y, Yuan K, et al. Influence of acupuncture treatment on cerebral activity in functional dyspepsia patients and its relationship with efficacy. Am J Gastroenterol. 2012;107:1236-47.
248. Schliessbach J, van der Klift E, Siegenthaler A, Arendt-Nielsen L, Curatolo M, Streitberger K. Does acupuncture needling induce analgesic effects comparable to diffuse noxious inhibitory controls? Evid Based Complement Alternat Med. 2012;2012:785613.
249. Wu S, Yamaguchi H, Shibutani K. Effect of acupuncture on perception threshold: a randomised controlled trial. Acupunct Med. 2012;30:32-6.
250. Akbar N, Zubieta J, Love T, et al. Differential acute effects of real and sham acupuncture on mu-opioid receptor availability in treatment naive and conditioned chronic pain patients. BMC Complementary and Alternative Medicine. 2012;12:O30.
251. do Prado JM, Kurebayashi LF, da Silva MJ. Efficacy of auriculotherapy for the reduction of stress in nursing students: a randomized clinical trial. Rev Lat Am Enfermagem. 2012;20:727-35. English, Portuguese, Spanish.
252. Quispe-Cabanillas JG, Damasceno A, von Glehn F, Brandão CO, Damasceno BP, Silveira WD, et al. Impact of electroacupuncture on quality of life for patients with Relapsing-Remitting Multiple Sclerosis under treatment with immunomodulators: a randomized study. BMC Complement Altern Med. 2012;12:209.
253. Ma TT, Yu SY, Li Y, Liang FR, Tian XP, Zheng H, et al. Randomised clinical trial: an assessment of acupuncture on specific meridian or specific acupoint vs. sham acupuncture for treating functional dyspepsia. Aliment Pharmacol Ther. 2012;35:552-61.
254. Rebhorn C, Breimhorst M, Buniatyan D, Vogel C, Birklein F, Eberle T. The efficacy of acupuncture in human pain models: a randomized, controlled, double-blinded study. Pain. 2012;153:1852-1862.
255. Abdi H, Zhao B, Darbandi M, Ghayour-Mobarhan M, Tavallaie S, Rahsepar AA, et al. The effects of body acupuncture on obesity: anthropometric parameters, lipid profile, and inflammatory and immunologic markers. Scientific World Journal. 2012;2012:603539.
256. White P, Bishop FL, Prescott P, Scott C, Little P, Lewith G. Practice, practitioner, or placebo? A multifactorial, mixed-methods randomized controlled trial of acupuncture. Pain. 2012;153:455-462.
257. Chan SL, Or KH, Sun WZ, Ng KY, Lo SK, Lee YS. Therapeutic effects of acupuncture for neurogenic dysphagia--a randomized controlled trial. J Tradit Chin Med. 2012;32:25-30.
258. Xu M, Zhou SJ, Jiang CC, Wu Y, Shi WL, Gu HH, et al. The effects of P6 electrical acustimulation on postoperative nausea and vomiting in patients after infratentorial craniotomy. J Neurosurg Anesthesiol. 2012;24:312-6.
259. Langenbach MR, Aydemir-Dogruyol K, Issel R, Sauerland S. Randomized sham-controlled trial of acupuncture for postoperative pain control after stapled haemorrhoidopexy. Colorectal Dis. 2012;14:e486-91.
260. Yao E, Gerritz PK, Henricson E, Abresch T, Kim J, Han J, et al. Randomized controlled trial comparing acupuncture with placebo acupuncture for the treatment of carpal tunnel syndrome. PM R. 2012;4:367-73.
261. Witt CM, Meissner K, Pach D, Thiele C, Lüdtke R, Ghadiyali Z,et al. Stimulation of gastric slow waves with manual acupuncture at acupuncture points ST36 and PC6--a randomized single blind controlled trial. Neurogastroenterol Motil. 2012;24:438-45, e211-2.
262. Chan SL, Or KH, Sun WZ, Ng KY, Lo SK, Lee YS. Therapeutic effects of acupuncture for neurogenic dysphagia--a randomized controlled trial. J Tradit Chin Med. 2012;32:25-30.
263. Suzuki M, Muro S, Ando Y, Omori T, Shiota T, Endo K, et al. A randomized, placebo-controlled trial of acupuncture in patients with chronic obstructive pulmonary disease (COPD): the COPD-acupuncture trial (CAT). Arch Intern Med. 2012;172:878-86.
264. Kim HM, Cho SY, Park SU, Sohn IS, Jung WS, Moon SK, et al. Can acupuncture affect the circadian rhythm of blood pressure? A randomized, double-blind, controlled trial. J Altern Complement Med. 2012;18:918-23.
265. Enblom A, Johnsson A, Hammar M, Onelöv E, Steineck G, Börjeson S. Acupuncture compared with placebo acupuncture in radiotherapy-induced nausea--a randomized controlled study. Ann Oncol. 2012;23:1353-1361.
266. Huang Y, Tang C, Wang S, Lu Y, Shen W, Yang J, et al. Acupuncture regulates the glucose metabolism in cerebral functional regions in chronic stage ischemic stroke patients--a PET-CT cerebral functional imaging study. BMC Neurosci. 2012;13:75.
267. Shen PF, Kong L, Ni LW, Guo HL, Yang S, Zhang LL, et al. Acupuncture intervention in ischemic stroke: a randomized controlled prospective study. Am J Chin Med. 2012;40:685-93.
268. Hsing WT, Imamura M, Weaver K, Fregni F, Azevedo Neto RS. Clinical effects of scalp electrical acupuncture in stroke: a sham-controlled randomized clinical trial. J Altern Complement Med. 2012;18:341-6.
269. Wallasch TM, Weinschuetz T, Mueller B, Kropp P. Cerebrovascular response in migraineurs during prophylactic treatment with acupuncture: a randomized controlled trial. J Altern Complement Med. 2012;18:777-83.
270. Chen J, Sarosiek I, Mccallum R, et al. Chronic electrical stimulation at acupuncture points improves dyspeptic symptoms in patients with diabetic gastroparesis: Official journal of the American College of Gastroenterology. 2012; doi:10.14309/00000434-201210001-00135.
271. Enblom A, Johnsson A, Hammar M, Onelöv E, Steineck G, Börjeson S. Acupuncture compared with placebo acupuncture in radiotherapy-induced nausea--a randomized controlled study. Ann Oncol. 2012;23:1353-1361.
272. Napadow V, Li A, Loggia M, et al. Brain circuitry subserving acupuncture relief of itch in atopic dermatitis: an fMRI study. Bmc Complementary & Alternative Medicine. 2012; doi:10.1186/1472-6882-12-S1-O32.
273. Huang Y, Tang C, Wang S, Lu Y, Shen W, Yang J, et al. Acupuncture regulates the glucose metabolism in cerebral functional regions in chronic stage ischemic stroke patients--a PET-CT cerebral functional imaging study. BMC Neurosci. 2012;13:75.
274. Liang H, Qu J. Decreased incidence of SIRS and sepsis by acupuncture in severe multiple traumatic patients via facilitation of vagal activity. Critical Care. 2012; doi:10.1186/cc11725.
275. Amos Z, Yoav M, Guy A, et al. A randomised controlled trial of an integrative approach utilising acupuncture for back and neck pain in an emergency department setting. European Journal of Integrative Medicine. 2012;4(supp-S1):23-24.
276. Mehta P K, Polk D, Shufelt C, et al. A randomized controlled trial of autonomic remodeling by sympathovagal modulation in coronary heart disease. Journal of the American College of Cardiology. 2012;59:E1775.
277. Chen JR, Li GL, Zhang GF, Huang Y, Wang SX, Lu N. Brain areas involved in acupuncture needling sensation of de qi: a single-photon emission computed tomography (SPECT) study. Acupunct Med. 2012;30:316-23.
278. Ng SS, Leung WW, Hon SS, Li JC, Wong CY, Lee JF. Electroacupuncture for ileus after laparoscopic colorectal surgery: a randomised sham-controlled study. Hong Kong Med J. 2013;19 Suppl 9:33-5.
279. Elseify MY, Mohammed NH, Alsharkawy AA, Elseoudy ME. Laser acupuncture in treatment of childhood bronchial asthma. J Complement Integr Med. 2013; doi: 10.1515/jcim-2012-0006.
280. Ajori L, Nazari L, Eliaspour D. Effects of acupuncture for initiation of labor: a double-blind randomized sham-controlled trial. Arch Gynecol Obstet. 2013;287:887-91.
281. Li G, Li S, An L, Wang B. Electroacupuncture alleviates intraoperative immunosuppression in patients undergoing supratentorial craniotomy. Acupunct Med. 2013;31:51-6.
282. Carvalho F, Weires K, Ebling M, Padilha Mde S, Ferrão YA, Vercelino R. Effects of acupuncture on the symptoms of anxiety and depression caused by premenstrual dysphoric disorder. Acupunct Med. 2013;31:358-63.
283. Darbandi S, Darbandi M, Mokarram P, Owji AA, Zhao B, Ghayor-Mobarhan M, et al. Effects of body electroacupuncture on plasma leptin concentrations in obese and overweight people in Iran: a randomized controlled trial. Altern Ther Health Med. 2013;19:24-31.
284. Villahermosa DI, Santos LG, Nogueira MB, Vilarino FL, Barbosa CP. Influence of acupuncture on the outcomes of in vitro fertilisation when embryo implantation has failed: a prospective randomised controlled clinical trial. Acupunct Med. 2013;31:157-61.
285. Yu DT, Jones AY. Physiological changes associated with de qi during electroacupuncture to LI4 and LI11: a randomised, placebo-controlled trial. Acupunct Med. 2013;31:143-50.
286. Paulson KL, Shay BL. Sympathetic nervous system responses to acupuncture and non-penetrating sham acupuncture in experimental forearm pain: a single-blind randomised descriptive study. Acupunct Med. 2013;31:178-84.
287. Karner M, Brazkiewicz F, Remppis A, Fischer J, Gerlach O, Stremmel W, et al. Objectifying specific and nonspecific effects of acupuncture: a double-blinded randomised trial in osteoarthritis of the knee. Evid Based Complement Alternat Med. 2013;2013:427265.
288. Brinkhaus B, Ortiz M, Witt CM, Roll S, Linde K, Pfab F, et al. Acupuncture in patients with seasonal allergic rhinitis: a randomized trial. Ann Intern Med. 2013;158:225-34.
289. Choi SM, Park JE, Li SS, Jung H, Zi M, Kim TH, et al. A multicenter, randomized, controlled trial testing the effects of acupuncture on allergic rhinitis. Allergy. 2013;68:365-74.
290. Bao T, Cai L, Giles JT, Gould J, Tarpinian K, Betts K, et al. A dual-center randomized controlled double blind trial assessing the effect of acupuncture in reducing musculoskeletal symptoms in breast cancer patients taking aromatase inhibitors. Breast Cancer Res Treat. 2013;138:167-74.
291. Deng G, Wong WD, Guillem J, Chan Y, Affuso T, Yeung KS, et al. A phase II, randomized, controlled trial of acupuncture for reduction of Postcolectomy Ileus. Ann Surg Oncol. 2013;20:1164-9.
292. Spaeth RB, Camhi S, Hashmi JA, Vangel M, Wasan AD, Edwards RR, et al. A longitudinal study of the reliability of acupuncture deqi sensations in knee osteoarthritis. Evid Based Complement Alternat Med. 2013;2013:204259.
293. Hachul H, Garcia TK, Maciel AL, Yagihara F, Tufik S, Bittencourt L. Acupuncture improves sleep in postmenopause in a randomized, double-blind, placebo-controlled study. Climacteric. 2013;16:36-40.
294. Bokmand S, Flyger H. Acupuncture relieves menopausal discomfort in breast cancer patients: a prospective, double blinded, randomized study. Breast. 2013;22:320-3.
295. Hasan SI, Mustafa M, Rashid RA, et al. 2692 – A study on the efectiveness of electroacupuncture in the management of sleep disorder among methadone maintenance therapy patients in kajang: a preliminary investigation. European Psychiatry. 2013;28(Suppl 1):1-1.
296. Ferreira LA, de Oliveira RG, Guimarães JP, Carvalho AC, De Paula MV. Laser acupuncture in patients with temporomandibular dysfunction: a randomized controlled trial. Lasers Med Sci. 2013;28:1549-58.
297. Chen H, Liu TY, Kuai L, Zhu J, Wu CJ, Liu LM. Electroacupuncture treatment for pancreatic cancer pain: a randomized controlled trial. Pancreatology. 2013;13:594-7.
298. Ng SSM, Leung WW, Mak TWC, Hon SSF, Li JCM, Wong CYN, et al. Electroacupuncture reduces duration of postoperative ileus after laparoscopic surgery for colorectal cancer. Gastroenterology. 2013;144:307-313.e1.
299. Quah-Smith I, Smith C, Crawford JD, Russell J. Laser acupuncture for depression: a randomised double blind controlled trial using low intensity laser intervention. J Affect Disord. 2013;148:179-87.
300. Zhang SP, Chiu TT, Chiu SN. Long-term efficacy of electroacupuncture for chronic neck pain: a randomised controlled trial. Hong Kong Med J. 2013;19 Suppl 9:36-9.
301. Ng SS, Leung WW, Chan SK, Wong CY, Lee J. Electroacupuncture analgesia for colonoscopy: a prospective, randomized, sham-controlled study. Gastrointestinal Endoscopy. 2013; doi:10.1016/j.gie.2013.04.140.
302. Liu Y, Liu L, Wang X. Electroacupuncture at points Baliao and Huiyang (BL35) for post-stroke detrusor overactivity. Neural Regen Res. 2013;8:1663-72.
303. Chen CY, Ke MD, Kuo CD, Huang CH, Hsueh YH, Chen JR. The influence of electro-acupuncture stimulation to female constipation patients. Am J Chin Med. 2013;41:301-13.
304. Zhang ZJ, Ng R, Man SC, Li JT, Wong W, Wong HK, et al. Use of electroacupuncture to accelerate the antidepressant action of selective serotonin reuptake inhibitors: a single-blind, randomised, controlled study. Hong Kong Med J. 2013;19 Suppl 9:12-6.
305. Vase L, Baram S, Takakura N, Yajima H, Takayama M, Kaptchuk TJ, et al. Specifying the nonspecific components of acupuncture analgesia. Pain. 2013;154:1659-1667.
306. Kang OS, Kim SY, Jahng GH, Kim H, Kim JW, Chung SY, et al. Neural substrates of acupuncture in the modulation of cravings induced by smoking-related visual cues: an fMRI study. Psychopharmacology (Berl). 2013;228:119-27.
307. Li G, Li S, Wang B, An L. The effect of electroacupuncture on postoperative immunoinflammatory response in patients undergoing supratentorial craniotomy. Exp Ther Med. 2013;6:699-702.
308. Harte SE, Clauw DJ, Napadow V, Harris RE. Pressure pain sensitivity and insular combined glutamate and glutamine (Glx) are associated with subsequent clinical response to sham but not traditional acupuncture in patients who have chronic pain. Med Acupunct. 2013;25:154-160.
309. Leung WW, Jones AY, Ng SS, Wong CY, Lee JF. Acupuncture transcutaneous electrical nerve stimulation reduces discomfort associated with barostat-induced rectal distension: a randomized-controlled study. World J Gastroenterol. 2013;19:381-8.
310. Maeda Y, Kettner N, Lee J, Kim J, Cina S, Malatesta C, et al. Acupuncture-evoked response in somatosensory and prefrontal cortices predicts immediate pain reduction in carpal tunnel syndrome. Evid Based Complement Alternat Med. 2013;2013:795906.
311. Lan L, Gao Y, Zeng F, Qin W, Dong M, Liu M, et al. A central analgesic mechanism of acupuncture for migraine: An ongoing functional MRI study. Neural Regen Res. 2013;8:2649-55.
312. Zhang J, Wang X, Lü R. Analgesic effect of acupuncture at hegu (LI 4) on transvaginal oocyte retrieval with ultrasonography. J Tradit Chin Med. 2013;33:294-7.
313. Deng G, Chan Y, Sjoberg D, Vickers A, Yeung KS, Kris M. Acupuncture for the treatment of post-chemotherapy chronic fatigue: a randomized, blinded, sham-controlled trial. Support Care Cancer. 2013;21:1735-41.
314. Cho YJ, Song YK, Cha YY, Shin BC, Shin IH, Park HJ, et al. Acupuncture for chronic low back pain: a multicenter, randomized, patient-assessor blind, sham-controlled clinical trial. Spine (Phila Pa 1976). 2013;38:549-57.
315. Kim EJ, Lim CY, Lee EY, Lee SD, Kim KS. Comparing the effects of individualized, standard, sham and no acupuncture in the treatment of knee osteoarthritis: a multicenter randomized controlled trial. Trials. 2013;14:129.
316. Tatiana, Molinas, Hasegawa, et al. Acupuncture for acute non-specific low back pain: a randomised, controlled, double-blind, placebo trial[J]. Acupuncture in medicine : journal of the British Medical Acupuncture Society. 2014;32:109-115.
317. Lin RT, Pai HC, Lee YC, Tzeng CY, Chang CH, Hung PH, et al. Electroacupuncture and rosiglitazone combined therapy as a means of treating insulin resistance and type 2 diabetes mellitus: a randomized controlled trial. Evid Based Complement Alternat Med. 2013;2013:969824.
318. Chung YC, Chien HC, Chen HH, Yeh ML. Acupoint stimulation to improve analgesia quality for lumbar spine surgical patients. Pain Manag Nurs. 2014;15:738-47.
319. Dias M, Vellarde GC, Olej B, Teófilo Salgado AE, de Barros Rezende I. Effects of electroacupuncture on stress-related symptoms in medical students: a randomised placebo-controlled study. Acupunct Med. 2014;32:4-11.
320. Yu JB, Dong SA, Gong LR, Wang M, Mu R, Li C. Effect of electroacupuncture at Zusanli (ST36) and Sanyinjiao (SP6) acupoints on adrenocortical function in etomidate anesthesia patients. Med Sci Monit. 2014;20:406-12.
321. Yang M, Yang J, Zeng F, Liu P, Lai Z, Deng S, et al. Electroacupuncture stimulation at sub-specific acupoint and non-acupoint induced distinct brain glucose metabolism change in migraineurs: a PET-CT study. J Transl Med. 2014;12:351.
322. Li H, Liu H, Liu C, Shi G, Zhou W, Zhao C, et al. Effect of "deqi" during the study of needling "Wang's Jiaji" acupoints treating spasticity after stroke. Evid Based Complement Alternat Med. 2014;2014:715351.
323. Shokrani O, Saghaei M, Ashrafi F, Sadeghi A. Electrical stimulation of acupuncture points for analgesia during bone marrow aspiration and biopsy: a randomized double-blind placebo-controlled trial. Adv Biomed Res. 2014;3:125.
324. Al Rashoud AS, Abboud RJ, Wang W, Wigderowitz C. Efficacy of low-level laser therapy applied at acupuncture points in knee osteoarthritis: a randomised double-blind comparative trial. Physiotherapy. 2014;100:242-8.
325. Xie YH, Chai XQ, Wang YL, Gao YC, Ma J. Effect of electro-acupuncture stimulation of Ximen (PC4) and Neiguan (PC6) on remifentanil-induced breakthrough pain following thoracal esophagectomy. J Huazhong Univ Sci Technolog Med Sci. 2014;34:569-574.
326. Mao JJ, Farrar JT, Bruner D, Zee J, Bowman M, Seluzicki C, et al. Electroacupuncture for fatigue, sleep, and psychological distress in breast cancer patients with aromatase inhibitor-related arthralgia: a randomized trial. Cancer. 2014;120:3744-51.
327. Hervik J, Mjåland O. Long term follow up of breast cancer patients treated with acupuncture for hot flashes. Springerplus. 2014;3:141.
328. Au-Yeung SS, Hui-Chan CW. Electrical acupoint stimulation of the affected arm in acute stroke: a placebo-controlled randomized clinical trial. Clin Rehabil. 2014;28:149-58.
329. Ntritsou V, Mavrommatis C, Kostoglou C, Dimitriadis G, Tziris N, Zagka P, et al. Effect of perioperative electroacupuncture as an adjunctive therapy on postoperative analgesia with tramadol and ketamine in prostatectomy: a randomised sham-controlled single-blind trial. Acupunct Med. 2014;32:215-22.
330. Zhang X, Jin HF, Fan YH, Lu B, Meng LN, Chen JD. Effects and mechanisms of transcutaneous electroacupuncture on chemotherapy-induced nausea and vomiting. Evid Based Complement Alternat Med. 2014;2014:860631.
331. Zhang Z, Wang C, Li Q, Zhang M, Zhao H, Dong L, et al. Electroacupuncture at ST36 accelerates the recovery of gastrointestinal motility after colorectal surgery: a randomised controlled trial. Acupunct Med. 2014;32:223-6.
332. Teoh A Y, Leung W W, Chong C, et al. Electroacupuncture analgesia for endoscopic ultrasound: A prospective, randomized, double-blinded, sham-controlled study. Gastroenterology. 2014;146:S-902.
333. Bauml J, Xie SX, Farrar JT, Bowman MA, Li SQ, Bruner D, et al. Expectancy in real and sham electroacupuncture: does believing make it so? J Natl Cancer Inst Monogr. 2014;2014:302-7.
334. Wilke J, Vogt L, Niederer D, Hübscher M, Rothmayr J, Ivkovic D, et al. Short-term effects of acupuncture and stretching on myofascial trigger point pain of the neck: a blinded, placebo-controlled RCT. Complement Ther Med. 2014;22:835-41.
335. Glazov G, Yelland M, Emery J. Low-dose laser acupuncture for non-specific chronic low back pain: a double-blind randomised controlled trial. Acupunct Med. 2014;32:116-23.
336. Zotelli VL, Grillo CM, de Sousa Mda L. Nausea control by needling at acupuncture point Neiguan (PC6) during an intraoral impression-taking procedure. J Acupunct Meridian Stud. 2014;7:318-23.
337. Zhang Q, Gao Z, Wang H, Ma L, Guo F, Zhong H. The effect of pre-treatment with transcutaneous electrical acupoint stimulation on the quality of recovery after ambulatory breast surgery: a prospective, randomised controlled trial. Anaesthesia. 2014;69:832-9.
338. Wang H, Xie Y, Zhang Q, Xu N, Zhong H, Dong H, et al. Transcutaneous electric acupoint stimulation reduces intra-operative remifentanil consumption and alleviates postoperative side-effects in patients undergoing sinusotomy: a prospective, randomized, placebo-controlled trial. Br J Anaesth. 2014;112:1075-82.
339. Couto C, de Souza IC, Torres IL, Fregni F, Caumo W. Paraspinal stimulation combined with trigger point needling and needle rotation for the treatment of myofascial pain: a randomized sham-controlled clinical trial. Clin J Pain. 2014 Mar;30:214-23.
340. Foroughipour M, Golchian AR, Kalhor M, Akhlaghi S, Farzadfard MT, Azizi H. A sham-controlled trial of acupuncture as an adjunct in migraine prophylaxis. Acupunct Med. 2014;32:12-6.
341. Aydoğmuş Y, Sunay M, Arslan H, Aydın A, Adiloğlu AK, Şahin H. Acupuncture versus solifenacin for treatment of overactive bladder and its correlation with urine nerve growth factor levels: a randomized, placebo-controlled clinical trial. Urol Int. 2014;93:437-43.
342. Lee SW, Liong ML, Yuen KH, Krieger JN. Acupuncture and immune function in chronic prostatitis/chronic pelvic pain syndrome: a randomized, controlled study. Complement Ther Med. 2014;22:965-9.
343. Hinman RS, McCrory P, Pirotta M, Relf I, Forbes A, Crossley KM, et al. Acupuncture for chronic knee pain: a randomized clinical trial. JAMA. 2014;312:1313-22.
344. Chen WT, Chang FC, Chen YH, Lin JG. An Evaluation of electroacupuncture at the Weizhong acupoint (BL-40) as a means of relieving pain induced by extracorporeal shock wave lithotripsy. Evid Based Complement Alternat Med. 2014;2014:592319.
345. Mehta PK, Polk DM, Zhang X, Li N, Painovich J, Kothawade K, et al. A randomized controlled trial of acupuncture in stable ischemic heart disease patients. Int J Cardiol. 2014;176:367-74.
346. Qi J, Chen J, Huang Y, Lai X, Tang C, Yang J, et al. Acupuncture at Waiguan (SJ5) and sham points influences activation of functional brain areas of ischemic stroke patients: a functional magnetic resonance imaging study. Neural Regen Res. 2014;9:293-300.
347. Yi WM, Chen Q, Liu CH, Hou JY, Chen LD, Wu WK. Acupuncture for preventing complications after radical hysterectomy: a randomized controlled clinical trial. Evid Based Complement Alternat Med. 2014;2014:802134.
348. Shan Y, Wang ZQ, Zhao ZL, Zhang M, Hao SL, Xu JY, et al. An FMRI study of neuronal specificity in acupuncture: the multiacupoint siguan and its sham point. Evid Based Complement Alternat Med. 2014;2014:103491.
349. Mao JJ, Xie SX, Farrar JT, Stricker CT, Bowman MA, Bruner D, et al. A randomised trial of electro-acupuncture for arthralgia related to aromatase inhibitor use. Eur J Cancer. 2014;50:267-76.
350. Ortiz M, Witt CM, Binting S, Helmreich C, Hummelsberger J, Pfab F, et al. A randomised multicentre trial of acupuncture in patients with seasonal allergic rhinitis--trial intervention including physician and treatment characteristics. BMC Complement Altern Med. 2014;14:128.
351. Chan YY, Lo WY, Li TC, Shen LJ, Yang SN, Chen YH, et al. Clinical efficacy of acupuncture as an adjunct to methadone treatment services for heroin addicts: a randomized controlled trial. Am J Chin Med. 2014;42:569-86.
352. Cafaro A, Arduino PG, Gambino A, Romagnoli E, Broccoletti R. Effect of laser acupuncture on salivary flow rate in patients with Sjögren's syndrome. Lasers Med Sci. 2015;30:1805-9.
353. Shafiei B, Heshmatipour M, Tavakol S, Saghaei M, Ghayumi Z. Determining the effect of laser acupuncture in treating stutterers in comparison with speech therapy. Adv Biomed Res. 2015;4:8.
354. Mao JJ, Bowman MA, Xie SX, Bruner D, DeMichele A, Farrar JT. Electroacupuncture versus gabapentin for hot flashes among breast cancer survivors: a randomized placebo-controlled trial. J Clin Oncol. 2015;33:3615-20.
355. Asadi N, Maharlouei N, Khalili A, Darabi Y, Davoodi S, Raeisi Shahraki H, et al. Effects of LI-4 and SP-6 acupuncture on labor pain, cortisol level and duration of labor. J Acupunct Meridian Stud. 2015;8:249-54.
356. López-Garrido B, García-Gonzalo J, Patrón-Rodriguez C, Marlasca-Gutiérrez MJ, Gil-Pita R, Toro-Flores R. Influence of acupuncture on the third stage of labor: a randomized controlled trial. J Midwifery Womens Health. 2015;60:199-205.
357. Zhang J, Cheng W, Cai M. Effects of electroacupuncture on overactive bladder refractory to anticholinergics: a single-blind randomised controlled trial. Acupunct Med. 2015;33:368-74.
358. Zhao W, Wang C, Li Z, Chen L, Li J, Cui W, et al. Efficacy and safety of transcutaneous electrical acupoint stimulation to treat muscle spasticity following brain injury: a double-blinded, multicenter, randomized controlled trial. PLoS One. 2015;10:e0116976.
359. Wang Q, Liang D, Wang F, Li W, Han Y, Zhang W, et al. Efficacy of electroacupuncture pretreatment for myocardial injury in patients undergoing percutaneous coronary intervention: a randomized clinical trial with a 2-year follow-up. Int J Cardiol. 2015;194:28-35.
360. Lee JS, Kim SG, Jung TG, Jung WY, Kim SY. Effect of Zhubin (KI9) acupuncture in reducing alcohol craving in patients with alcohol dependence: a randomized placebo-controlled trial. Chin J Integr Med. 2015;21:307-11.
361. Zheng Y, Feng X, Mi H, Yao Y, Zhao Y, Li J, et al. Effects of transcutaneous electrical acupoint stimulation on ovarian reserve of patients with diminished ovarian reserve in in vitro fertilization and embryo transfer cycles. J Obstet Gynaecol Res. 2015;41:1905-11.
362. Liu X, Fan T, Lan Y, Dong S, Fu J, Mao B. Effects of transcutaneous electrical acupoint stimulation on patients with stable chronic obstructive pulmonary disease: a prospective, single-blind, randomized, placebo-controlled study. J Altern Complement Med. 2015;21:610-6.
363. Zhiyuan W, Ming Y, Jie J, Yi W, Tiansheng H, Mingfen L, et al. Effect of transcutaneous electrical nerve stimulation at acupoints on patients with type 2 diabetes mellitus: a randomized controlled trial. J Tradit Chin Med. 2015;35:134-40.
364. Gu XD, Wang J, Yu P, Li JH, Yao YH, Fu JM, et al. Effects of electroacupuncture combined with clean intermittent catheterization on urinary retention after spinal cord injury: a single blind randomized controlled clinical trial. Int J Clin Exp Med. 2015;8:19757-63.
365. Shuai Z, Lian F, Li P, Yang W. Effect of transcutaneous electrical acupuncture point stimulation on endometrial receptivity in women undergoing frozen-thawed embryo transfer: a single-blind prospective randomised controlled trial. Acupunct Med. 2015;33:9-15.
366. Liu X, Li S, Wang B, An L, Ren X, Wu H. Intraoperative and postoperative anaesthetic and analgesic effect of multipoint transcutaneous electrical acupuncture stimulation combined with sufentanil anaesthesia in patients undergoing supratentorial craniotomy. Acupunct Med. 2015;33:270-6.
367. Shin JY, Ku B, Kim JU, Lee YJ, Kang JH, Heo H, et al. Short-term effect of laser acupuncture on lower back pain: a randomized, placebo-controlled, double-blind trial. Evid Based Complement Alternat Med. 2015;2015:808425.
368. Chen Y, Wu W, Yao Y, Yang Y, Zhao Q, Qiu L. Transcutaneous electric acupoint stimulation at Jiaji points reduce abdominal pain after colonoscopy: a randomized controlled trial. Int J Clin Exp Med. 2015;8:5972-7.
369. Albrecht T, Wu S, Baumann I, Plinkert PK, Sertel S. Measurable impact of acupuncture on mucosal swelling of inferior turbinates: a prospective, randomized, controlled study. Acta Otolaryngol. 2015;135:169-76.
370. Aranha MF, Müller CE, Gavião MB. Pain intensity and cervical range of motion in women with myofascial pain treated with acupuncture and electroacupuncture: a double-blinded, randomized clinical trial. Braz J Phys Ther. 2015;19:34-43.
371. Qian X, Zhou X, You Y, Shu S, Fang F, Huang S, et al. Traditional Chinese acupuncture for poststroke depression: a single-blind double-simulated randomized controlled trial. J Altern Complement Med. 2015;21:748-53.
372. Chen X, Spaeth RB, Freeman SG, Scarborough DM, Hashmi JA, Wey HY, et al. The modulation effect of longitudinal acupuncture on resting state functional connectivity in knee osteoarthritis patients. Mol Pain. 2015;11:67.
373. Dalamagka M, Mavrommatis C, Grosomanidis V, Karakoulas K, Vasilakos D. Postoperative analgesia after low-frequency electroacupuncture as adjunctive treatment in inguinal hernia surgery with abdominal wall mesh reconstruction. Acupunct Med. 2015;33:360-7.
374. Shen Y, Liu L, Chiang JS, Meng Z, Garcia MK, Chen Z, et al. Randomized, placebo-controlled trial of K1 acupoint acustimulation to prevent cisplatin-induced or oxaliplatin-induced nausea. Cancer. 2015;121:84-92.
375. Yao Y, Zhao Q, Gong C, Wu Y, Chen Y, Qiu L, et al. Transcutaneous electrical acupoint stimulation improves the postoperative quality of recovery and analgesia after gynecological laparoscopic surgery: a randomized controlled trial. Evid Based Complement Alternat Med. 2015;2015:324360.
376. Chen Y, Yao Y, Wu Y, Dai D, Zhao Q, Qiu L. Transcutaneous electric acupoint stimulation alleviates remifentanil-induced hyperalgesia in patients undergoing thyroidectomy: a randomized controlled trial. Int J Clin Exp Med. 2015;8:5781-7.
377. Zuppa C, Prado CH, Wieck A, Zaparte A, Barbosa A, Bauer ME. Acupuncture for sleep quality, BDNF levels and immunosenescence: a randomized controlled study. Neurosci Lett. 2015;587:35-40.
378. Wang Y, Xue CC, Helme R, Da Costa C, Zheng Z. Acupuncture for frequent migraine: a randomized, patient/assessor blinded, controlled trial with one-year follow-up. Evid Based Complement Alternat Med. 2015;2015:920353.
379. Chung KF, Yeung WF, Yu YM, Yung KP, Zhang SP, Zhang ZJ, et al. Acupuncture for residual insomnia associated with major depressive disorder: a placebo- and sham-controlled, subject- and assessor-blind, randomized trial. J Clin Psychiatry. 2015;76:e752-60.
380. Zheng H, Huang W, Li J, Zheng Q, Li Y, Chang X, et al. Association of pre- and post-treatment expectations with improvements after acupuncture in patients with migraine. Acupunct Med. 2015;33:121-8.
381. Zhu B, Wang Y, Zhang G, Ouyang H, Zhang J, Zheng Y, et al. Acupuncture at KI3 in healthy volunteers induces specific cortical functional activity: an fMRI study. BMC Complement Altern Med. 2015;15:361.
382. Swanson B, Keithley JK, Johnson A, Fogg L, Adeyemi O, Sha BE, et al. Acupuncture to reduce HIV-associated inflammation. Evid Based Complement Alternat Med. 2015;2015:908538.
383. Gemma M, Nicelli E, Gioia L, Moizo E, Beretta L, Calvi MR. Acupuncture accelerates recovery after general anesthesia: a prospective randomized controlled trial. J Integr Med. 2015;13:99-104.
384. Sahin S, Bicer M, Eren GA, Tas S, Tugcu V, Tasci AI, et al. Acupuncture relieves symptoms in chronic prostatitis/chronic pelvic pain syndrome: a randomized, sham-controlled trial. Prostate Cancer Prostatic Dis. 2015;18:249-54.
385. Zheng Y, Wang Y, Lan Y, Qu X, Lin K, Zhang J, et al. Imaging of brain function based on the analysis of functional connectivity- Imaging analysis of brain function by fMRI after acupuncture at LR3 in healthy individuals. Afr J Tradit Complement Altern Med. 2016;13:90-100.
386. McDonald JL, Smith PK, Smith CA, Changli Xue C, Golianu B, Cripps AW, et al. Effect of acupuncture on house dust mite specific IgE, substance P, and symptoms in persistent allergic rhinitis. Ann Allergy Asthma Immunol. 2016;116:497-505.
387. Yiu EM, Chan KM, Kwong E, Li NY, Ma EP, Tse FW, et al. Is Acupuncture efficacious for treating phonotraumatic vocal pathologies? A randomized control trial. J Voice. 2016;30:611-20.
388. Bilici M, Güven S, Köşker S, Şafak A, Semiz ÜB. Electroacupuncture Therapy in nicotine dependence: a double blind, sham-controlled study. Noro Psikiyatr Ars. 2016;53:28-32.
389. Yeoh AH, Tang SS, Abdul Manap N, Wan Mat WR, Said S, Che Hassan MR, et al. Effectiveness of P6 acupoint electrical stimulation in preventing postoperativenausea and vomiting following laparoscopic surgery. Turk J Med Sci. 2016;46:620-5.
390. Chen T, Wang K, Xu J, Ma W, Zhou J. Electroacupuncture reduces postoperative pain and analgesic consumption in patients undergoing thoracic surgery: a randomized study. Evid Based Complement Alternat Med. 2016;2016:2126416.
391. Bittner A K, Seger K R, Kayser S, et al. Increased velocity of retinal blood flow in RP subjects with significantly improved visual function following transcorneal electrical stimulation in a randomized controlled trial. Investigative ophthalmology & visual science. 2016;(12):57.
392. da Graca-Tarragó M, Deitos A, Patrícia Brietzke A, Torres IL, Cadore Stefani L, Fregni F, et al. Electrical intramuscular stimulation in osteoarthritis enhances the inhibitory systems in pain processing at cortical and cortical spinal system. Pain Med. 2016;17:877-891.
393. Wik G, Huang Y, Zeng T, Qu S, Zheng Y, Zhang J, et al. Waiguan stimulation may kindle anticorrelated brain networks: functional magnetic resonance imaging data revisited. J Acupunct Meridian Stud. 2016;9:22-5.
394. Hung YC, Hung IL, Hu WL, Tseng YJ, Kuo CE, Liao YN, et al. Reduction in postpartum weight with laser acupuncture: a randomized control trial. Medicine (Baltimore). 2016;95:e4716.
395. Laureano MR, Onishi ET, Bressan RA, Neto PB, Castiglioni ML, Batista IR, et al. The effectiveness of acupuncture as a treatment for tinnitus: a randomized controlled trial using (99m)Tc-ECD SPECT. Eur Radiol. 2016;26:3234-42.
396. Feng B, Zhang ZJ, Zhu RM, Yuan GZ, Luo LY, McAlonan GM, et al. Transcutaneous electrical acupoint stimulation as an adjunct therapy for obsessive-compulsive disorder: a randomized controlled study. J Psychiatr Res. 2016;80:30-37.
397. Yiu EM, Chan KM, Li NY, Tsang R, Verdolini Abbott K, Kwong E, et al. Wound-healing effect of acupuncture for treating phonotraumatic vocal pathologies: A cytokine study. Laryngoscope. 2016;126:E18-22.
398. Hadadian F, Sohrabi N, Farokhpayam M, Farokhpayam H, Towhidi F, Fayazi S, et al. The effects of transcutaneous electrical acupoint stimulation (TEAS) on fatigue in haemodialysis patients. J Clin Diagn Res. 2016;10:YC01-YC04.
399. Kluger BM, Rakowski D, Christian M, Cedar D, Wong B, Crawford J, et al. Randomized, controlled trial of acupuncture for fatigue in Parkinson's disease. Mov Disord. 2016;31:1027-32.
400. Wang X, Wang Z, Liu J, Chen J, Liu X, Nie G, et al. Repeated acupuncture treatments modulate amygdala resting state functional connectivity of depressive patients. Neuroimage Clin. 2016;12:746-752.
401. Lee DH, Cho SY, Yang SB, Lee HM, Shin HS, Lee SH, et al. Efficacy of acupuncture treatment to prevent cerebral vasospasm after subarachnoid hemorrhage: a double-blind, randomized placebo-controlled trial. J Altern Complement Med. 2020;26:1182-1189.
402. Bilgin G, Arslan H, Balc N, et al. Acupuncture for the treatment of mild or moderate asthma: a randomized, placebo-controlled clinical trial. Nobel Medicus. 2016;12:31-37.
403. Lee GE, Son C, Lee J, et al. Acupuncture for shoulder pain after stroke: a randomized controlled clinical trial. European Journal of Integrative Medicine, 2016:373-383.
404. Li Z, Liu M, Lan L, Zeng F, Makris N, Liang Y, et al. Altered periaqueductal gray resting state functional connectivity in migraine and the modulation effect of treatment. Sci Rep. 2016;6:20298.
405. Huang Z, Zhang N, Xu F, Yin J, Dai N, Chen JD. Ameliorating effect of transcutaneous electroacupuncture on impaired gastric accommodation induced by cold meal in healthy subjects. J Gastroenterol Hepatol. 2016;31:561-6.
406. Sahin S, Bicer M, Yenice MG, Seker KG, Yavuzsan AH, Tugcu V. A prospective randomized controlled study to compare acupuncture and dapoxetine for the treatment of premature ejaculation. Urol Int. 2016;97:104-11.
407. Liu Z, Yan S, Wu J, He L, Li N, Dong G, et al. Acupuncture for chronic severe functional constipation: a randomized trial. Ann Intern Med. 2016;165:761-769.
408. Shayestehfar M, Seif-Barghi T, Zarei S, Mehran A. Acupuncture anxiolytic effects on physiological and psychological assessments for a clinical trial. Scientifica (Cairo). 2016;2016:4016952.
409. Feng J, Wang X, Li X, Zhao D, Xu J. Acupuncture for chronic obstructive pulmonary disease (COPD): A multicenter, randomized, sham-controlled trial. Medicine (Baltimore). 2016;95:e4879.
410. Rueda Garrido JC, Vas J, Lopez DR. Acupuncture treatment of shoulder impingement syndrome: a randomized controlled trial. Complement Ther Med. 2016;25:92-7.
411. Sokunbi G, Maduagwu S, Jaiyeola O, Gambo H, Blasu C. Cardiovascular response to manual acupuncture needle stimulation among apparently healthy nigerian adults. J Acupunct Meridian Stud. 2016;9:143-50.
412. Fan L, Fu W, Chen Z, Xu N, Liu J, Lü A, et al. Curative effect of acupuncture on quality of life in patient with depression: a clinical randomized single-blind placebo-controlled study. J Tradit Chin Med. 2016;36:151-9.
413. Zheng Y, Zhang J, Wang Y, Wang Y, Lan Y, Qu S, et al. Acupuncture decreases blood pressure related to hypothalamus functional connectivity with frontal lobe, cerebellum, and insula: a study of instantaneous and short-term acupuncture treatment in essential hypertension. Evid Based Complement Alternat Med. 2016;2016:6908710.
414. Helianthi DR, Simadibrata C, Srilestari A, Wahyudi ER, Hidayat R. Pain reduction after laser acupuncture treatment in geriatric patients with knee osteoarthritis: a randomized controlled trial. Acta Med Indones. 2016;48:114-21.
415. Fu C, Zhao N, Liu Z, Yuan LH, Xie C, Yang WJ, et al. Acupuncture improves peri-menopausal insomnia: a randomized controlled trial. Sleep. 2017;40(11).
416. Yin X, Gou M, Xu J, Dong B, Yin P, Masquelin F, et al. Efficacy and safety of acupuncture treatment on primary insomnia: a randomized controlled trial. Sleep Med. 2017;37:193-200.
417. Huang L, Pan Y, Chen S, Zhang M, Zhuang X, Jin S, et al. Prevention of propofol injection-related pain using pretreatment transcutaneous electrical acupoint stimulation. Turk J Med Sci. 2017;47:1267-1276.
418. Razavy S, Gadau M, Zhang SP, Wang FC, Bangrazi S, Berle C, et al. Investigation of the phenomenon of propagated sensation along the channels in the upper limb following administration of acupuncture and mock Laser. J Acupunct Meridian Stud. 2017;10:307-316.
419. Oates A, Benedict KA, Sun K, Brakeman PR, Lim J, Kim C. Laser acupuncture reduces pain in pediatric kidney biopsies: a randomized controlled trial. Pain. 2017;158:103-109.
420. Zucker NA, Tsodikov A, Mist SD, Cina S, Napadow V, Harris RE. Evoked pressure pain sensitivity is associated with differential analgesic response to verum and sham acupuncture in fibromyalgia. Pain Med. 2017;18:1582-1592.
421. Kibar S, Konak HE, Evcik D, Ay S. Laser acupuncture treatment improves pain and functional status in patients with subacromial impingement syndrome: a randomized, double-blind, sham-controlled study. Pain Med. 2017;18:980-987.
422. Schröder S, Meyer-Hamme G, Friedemann T, Kirch S, Hauck M, Plaetke R, et al. Immediate pain relief in adhesive capsulitis by acupuncture-a randomized controlled double-blinded study. Pain Med. 2017;18:2235-2247.
423. Schroeder S, Burnis J, Denton A, Krasnow A, Raghu TS, Mathis K. Effectiveness of acupuncture therapy on stress in a large urban college population. J Acupunct Meridian Stud. 2017;10:165-170.
424. Acosta-Olivo C, Siller-Adame A, Tamez-Mata Y, Vilchez-Cavazos F, Peña-Martinez V, Acosta-Olivo C. Laser treatment on acupuncture points improves pain and wrist functionality in patients undergoing rehabilitation therapy after wrist bone fracture. a randomized, controlled, blinded study. Acupunct Electrother Res. 2017;42:11-25.
425. Liu Z, Liu Y, Xu H, He L, Chen Y, Fu L, etal. Effect of electroacupuncture on urinary leakage among women with stress urinary incontinence: a randomized clinical trial. JAMA. 2017;317:2493-2501.
426. Xie J, Chen LH, Ning ZY, Zhang CY, Chen H, Chen Z, et al. Effect of transcutaneous electrical acupoint stimulation combined with palonosetron on chemotherapy-induced nausea and vomiting: a single-blind, randomized, controlled trial. Chin J Cancer. 2017;36:6.
427. Li M, Zhang B, Meng Z, Sha T, Han Y, Zhao H, et al. Effect of Tiaoshen Kaiqiao acupuncture in the treatment of ischemic post-stroke depression: a randomized controlled trial. J Tradit Chin Med. 2017;37:171-8.
428. Lin S, Wichai E, Amonrat J, Somchai R. Effect of acupuncture on heart rate variability during prolonged high-intensity training in soccer players. J Tradit Chin Med. 2017;37:636-642.
429. Widgren Y, Enblom A. Emesis in patients receiving acupuncture, sham acupuncture or standard care during chemo-radiation: a randomized controlled study. Complement Ther Med. 2017;34:16-25.
430. Fydanaki O, Kousoulis P, Dardiotis E, Bizakis I, Hajiioannou I. Electroacupuncture could reduce motion sickness susceptibility in healthy male adults: a double-blinded study. Med Acupunct. 2017;29:377-382.
431. Liu J, Ma S, Mu J, Chen T, Xu Q, Dun W, et al. Integration of white matter network is associated with interindividual differences in psychologically mediated placebo response in migraine patients. Hum Brain Mapp. 2017;38:5250-5259.
432. Huang S, Peng W, Tian X, Liang H, Jia Z, Lo T, et al. Effects of transcutaneous electrical acupoint stimulation at different frequencies on perioperative anesthetic dosage, recovery, complications, and prognosis in video-assisted thoracic surgical lobectomy: a randomized, double-blinded, placebo-controlled trial. J Anesth. 2017;31:58-65.
433. Zhou J, Yang L, Yu J, Wang Y, Liu Z. Efficacy of acupuncture on menstrual frequency in women with polycystic ovary syndrome: Protocol for a randomized, controlled trial. Medicine (Baltimore). 2017;96:e8828.
434. Mazidi M, Abbasi-Parizad P, Abdi H, Zhao B, Rahsepar AA, Tavallaie S, et al. The effect of electro-acupuncture on pro-oxidant antioxidant balance values in overweight and obese subjects: a randomized controlled trial study. J Complement Integr Med. 2017; doi: 10.1515/jcim-2015-0081.
435. Maeda Y, Kim H, Kettner N, Kim J, Cina S, Malatesta C, et al. Rewiring the primary somatosensory cortex in carpal tunnel syndrome with acupuncture. Brain. 2017;140:914-927.
436. Zhao L, Chen J, Li Y, Sun X, Chang X, Zheng H, et al. The long-term effect of acupuncture for migraine prophylaxis: a randomized clinical trial. JAMA Intern Med. 2017;177:508-515.
437. Öncü E, Zincir H. The effect of transcutaneous electrical nerve stimulation in patients with acute exacerbation of chronic obstructive pulmonary disease: randomised controlled trial. J Clin Nurs. 2017;26:1834-1844.
438. Tan TT, Wang D, Huang JK, Zhou XM, Yuan X, Liang JP, et al. Modulatory effects of acupuncture on brain networks in mild cognitive impairment patients. Neural Regen Res. 2017;12:250-258.
439. Enblom A, Steineck G, Hammar M, Börjeson S. Reduced need for rescue antiemetics and improved capacity to eat in patients receiving acupuncture compared to patients receiving sham acupuncture or standard care during radiotherapy. Evid Based Complement Alternat Med. 2017;2017:5806351.
440. Hou L, Zhou C, Wu Y, Yu Y, Hu Y. Transcutaneous electrical acupoint stimulation (TEAS) relieved cancer-related fatigue in non-small cell lung cancer (NSCLC) patients after chemotherapy. J Thorac Dis. 2017;9:1959-1966.
441. Li Z, Lan L, Zeng F, Makris N, Hwang J, Guo T, et al. The altered right frontoparietal network functional connectivity in migraine and the modulation effect of treatment. Cephalalgia. 2017;37:161-176.
442. Sun K, Xing T, Zhang F, Liu Y, Li W, Zhou Z, et al. Perioperative transcutaneous electrical acupoint stimulation for postoperative pain relief following laparoscopic surgery: a randomized controlled trial. Clin J Pain. 2017;33:340-347.
443. Tian L, Feng X, Zhang R, Wang S, Li R, Kong R, et al. Pain relief during oocyte retrieval by transcutaneous electrical acupoint stimulation: a single-blinded, randomized, controlled multicenter trial. Evid Based Complement Alternat Med. 2020;2020:3285648.
444. Ngai SP, Spencer LM, Jones AY, Alison JA. Acu-TENS reduces breathlessness during exercise in people with chronic obstructive pulmonary disease. Evid Based Complement Alternat Med. 2017;2017:3649257.
445. Wang Z, Wang X, Liu J, Chen J, Liu X, Nie G, et al. Acupuncture treatment modulates the corticostriatal reward circuitry in major depressive disorder. J Psychiatr Res. 2017;84:18-26.
446. Li Z, Zeng F, Yin T, Lan L, Makris N, Jorgenson K, et al. Acupuncture modulates the abnormal brainstem activity in migraine without aura patients. Neuroimage Clin. 2017;15:367-375.
447. Liao HY, Ho WC, Chen CC, Lin JG, Chang CC, Chen LY, et al. Clinical evaluation of acupuncture as treatment for complications of cerebrovascular accidents: a randomized, sham-controlled, subject- and assessor-blind trial. Evid Based Complement Alternat Med. 2017;2017:7498763.
448. Huang Y, Meng J, Sun B, Xiang T, Zhou X, Xu B, et al. Acupuncture for serum uric acid in patients with asymptomatic hyperuricemia: a randomized, double-blind, placebo-controlled trial. Int J Cardiol. 2017;232:227-232.
449. Zotelli VL, Grillo CM, Gil ML, Wada RS, Sato JE, da Luz Rosário de Sousa M. Acupuncture effect on pain, mouth opening limitation and on the energy meridians in patients with temporomandibular dysfunction: a randomized controlled trial. J Acupunct Meridian Stud. 2017;10:351-359.
450. Yin X, Gou M, Xu J, Dong B, Yin P, Masquelin F, et al. Efficacy and safety of acupuncture treatment on primary insomnia: a randomized controlled trial. Sleep Med. 2017;37:193-200.
451. Fu C, Zhao N, Liu Z, Yuan LH, Xie C, Yang WJ, et al. Acupuncture improves peri-menopausal insomnia: a randomized controlled trial. Sleep. 2017;40(11).
452. Nahidi Y, Badiee S, Torabi S, et al. Acupuncture effect on pruritus in hemodialysis patients: a randomized clinical trial. Iranian Red Crescent Medical Journal. 2018; doi:10.5812/ircmj.65521.
453. Armond ACV, Glória JCR, Dos Santos CRR, Galo R, Falci SGM. Acupuncture on anxiety and inflammatory events following surgery of mandibular third molars: a split-mouth, randomized, triple-blind clinical trial. Int J Oral Maxillofac Surg. 2019;48:274-281.
454. Mazda Y, Kikuchi T, Yoshimatsu A, Kato A, Nagashima S, Terui K. Acupuncture for reducing pruritus induced by intrathecal morphine at elective cesarean delivery: a placebo-controlled, randomized, double-blind trial. Int J Obstet Anesth. 2018;36:66-76.
455. Li Z, Chen J, Cheng J, Huang S, Hu Y, Wu Y, et al. Acupuncture modulates the cerebello-thalamo-cortical circuit and cognitive brain regions in patients of Parkinson's Disease with tremor. Front Aging Neurosci. 2018;10:206.
456. Liu Z, Ai Y, Wang W, Zhou K, He L, Dong G, et al. Acupuncture for symptoms in menopause transition: a randomized controlled trial. Am J Obstet Gynecol. 2018;219:373.e1-373.e10.
457. Deng G, Giralt S, Chung DJ, Landau H, Siman J, Search B, et al. Acupuncture for reduction of symptom burden in multiple myeloma patients undergoing autologous hematopoietic stem cell transplantation: a randomized sham-controlled trial. Support Care Cancer. 2018;26:657-665.
458. Razavy S, Gadau M, Zhang SP, Wang FC, Bangrazi S, Berle C, et al. Anxiety related to De Qi psychophysical responses as measured by MASS: a sub-study embedded in a multisite randomised clinical trial. Complement Ther Med. 2018;39:24-35.
459. Meyer-Hamme G, Friedemann T, Greten HJ, Plaetke R, Gerloff C, Schroeder S. ACUDIN - Acupuncture and laser acupuncture for treatment of diabetic peripheral neuropathy: a randomized, placebo-controlled, partially double-blinded trial. BMC Neurol. 2018;18:40.
460. Qin Z, Zang Z, Zhou K, Wu J, Zhou J, Kwong JSW, et al. Acupuncture for chronic prostatitis/chronic pelvic pain syndrome: a randomized, sham acupuncture controlled trial. J Urol. 2018;200:815-822.
461. Bittner AK, Seger K, Salveson R, Kayser S, Morrison N, Vargas P, et al. Randomized controlled trial of electro-stimulation therapies to modulate retinal blood flow and visual function in retinitis pigmentosa. Acta Ophthalmol. 2018;96:e366-e376.
462. Feiyi Z, Zheyuan Z, Yingxia Z, et al. The effect of electroacupuncture preconditioning on cognitive impairments following knee replacement among elderly: a randomized controlled trial. World Journal of Acupuncture - Moxibustion, 2018, 28:231-236.
463. Ketabchi AA, Salajegheh S. The effects of acupuncture treatment in infertile patients with clinical varicocele. Nephro-Urology Monthly. 2018; doi:10.5812/numonthly.65451.
464. Hershman D L, Unger J M, Greenlee H, et al. Effect of acupuncture vs sham acupuncture or waitlist control on joint pain related to aromatase inhibitors among women with early-stage breast cancer: a randomized clinical trial. JAMA The Journal of the American Medical Association, 2018, 320:167.
465. Karatay S, Okur SC, Uzkeser H, Yildirim K, Akcay F. Effects of acupuncture treatment on fibromyalgia symptoms, serotonin, and substance P levels: a randomized sham and placebo-controlled clinical trial. Pain Med. 2018;19:615-628.
466. Smith CA, de Lacey S, Chapman M, Ratcliffe J, Norman RJ, Johnson NP, et al. Effect of acupuncture vs sham acupuncture on live births among women undergoing in vitro fertilization: a randomized clinical trial. JAMA. 2018;319:1990-1998.
467. Zeng L, Tao Y, Hou W, Zong L, Yu L. Electro-acupuncture improves psychiatric symptoms, anxiety and depression in methamphetamine addicts during abstinence: a randomized controlled trial. Medicine (Baltimore). 2018;97:e11905.
468. Wu J, Chen B, Yin X, Yin P, Lao L, Xu S. Effect of acupuncture on post-hemorrhoidectomy pain: a randomized controlled trial. J Pain Res. 2018;11:1489-1496.
469. Mohammed N, Allam H, Elghoroury E, Zikri EN, Helmy GA, Elgendy A. Evaluation of serum beta-endorphin and substance P in knee osteoarthritis patients treated by laser acupuncture. J Complement Integr Med. 2018; doi: 10.1515/jcim-2017-0010.
470. Mayrink WC, Garcia JBS, Dos Santos AM, Nunes JKVRS, Mendonça THN. Effectiveness of acupuncture as auxiliary treatment for chronic headache. J Acupunct Meridian Stud. 2018;11:296-302.
471. Zheng H, Xu J, Sun X, Zeng F, Li Y, Wu X, et al. Electroacupuncture for patients with refractory functional dyspepsia: a randomized controlled trial. Neurogastroenterol Motil. 2018;30:e13316.
472. Teoh AYB, Chong CCN, Leung WW, Chan SKC, Tse YK, Ng EKW, et al. Electroacupuncture-reduced sedative and analgesic requirements for diagnostic EUS: a prospective, randomized, double-blinded, sham-controlled study. Gastrointest Endosc. 2018;87:476-485.
473. Fatemeh M, Ali K V, Atefeh S, et al. Efficacy of adding acupuncture to Methylphenidate in children and adolescents with attention deficit hyperactivity disorder: a randomized clinical trial. European Journal of Integrative Medicine. 2018;22:62-68.
474. AminiSaman J, Mohammadi S, Karimpour H, Hemmatpour B, Sharifi H, Kawyannejad R. Transcutaneous electrical nerve stimulation at the acupuncture points to relieve pain of patients under mechanical ventilation: a randomized controlled study. J Acupunct Meridian Stud. 2018;11:290-295.
475. Grillo CM, Zotelli VLR, Lúcia Bressiani Gil M, de Sousa MDLR. Would a placebo acupuncture needle be able to induce deqi? J Acupunct Meridian Stud. 2018;11:273-279.
476. Mi J, Chen X, Lin X, Guo J, Chen H, Wei L, et al. Treatment of persistent allergic rhinitis via acupuncture at the sphenopalatine acupoint: a randomized controlled trial. Trials. 2018;19:28.
477. Zhou D, Hu B, He S, Li X, Gong H, Li F, et al. Transcutaneous electrical acupoint stimulation accelerates the recovery of gastrointestinal function after cesarean section: a randomized controlled Trial. Evid Based Complement Alternat Med. 2018;2018:7341920.
478. Tu Q, Yang Z, Gan J, Zhang J, Que B, Song Q, et al. Transcutaneous electrical acupoint stimulation improves immunological function during the perioperative period in patients with non-small cell lung cancer undergoing video-assisted thoracic surgical lobectomy. Technol Cancer Res Treat. 2018; doi: 10.1177/1533033818806477.
479. Capodice JL, Parkhomenko E, Tran TY, Thai J, Blum KA, Chandhoke RA, et al. A Randomized, double-blind, sham-controlled study assessing electroacupuncture for the management of postoperative pain after Percutaneous Nephrolithotomy. J Endourol. 2019;33:194-200.
480. Tu Q, Gan J, Shi J, Yu H, He S, Zhang J. Effect of transcutaneous electrical acupoint stimulation on postoperative analgesia after ureteroscopic lithotripsy: a randomized controlled trial. Urolithiasis. 2019;47:279-287.
481. Zadeh B Q, Hadadian F, Salari N, et al. The effect of transcotaneus electrical acupoint stimulation on sleep quality in nurses[J]. 2018; doi:10.5812/jkums.68669.
482. Liu Z, Ge Y, Xu F, Xu Y, Liu Y, Xia F, et al. Preventive effects of transcutaneous electrical acustimulation on ischemic stroke-induced constipation mediated via the autonomic pathway. Am J Physiol Gastrointest Liver Physiol. 2018;315:G293-G301.
483. Qin Z, Zang Z, Zhou K, Wu J, Zhou J, Kwong JSW, et al. Acupuncture for chronic prostatitis/chronic pelvic pain syndrome: a randomized, sham acupuncture controlled trial. J Urol. 2018;200:815-822.
484. Ma G, Hu P, Zhang B, Xu F, Yin J, Yang X, et al. Transcutaneous electrical acustimulation synchronized with inspiration improves gastric accommodation impaired by cold stress in healthy subjects. Neurogastroenterol Motil. 2019;31:e13491.
485. Zhao FV. Attention network function of insomniacs improved by manual acupuncture: Evidence from Attention Network Task.World Journal of Acupuncture-Moxibustion,2019,29:91-96.
486. de Almeida TB, Zotelli VLR, Wada RS, Sousa MLR. Comparative analgesia between acupuncture and dipyrone in odontalgia. J Acupunct Meridian Stud. 2019;12:182-191.
487. Faiz SHR, Nikoubakht N, Imani F, Ziyaeifard M, Sadegh H, Rahimzadeh P. Comparison of two acupuncture protocols (K1, DU25 or K1, DU26) efficacy on recovery time of patients after general anesthesia, a randomized control clinical trial. Anesth Pain Med. 2019;9:e96172.
488. Zhao L, Li D, Zheng H, Chang X, Cui J, Wang R, et al. Acupuncture as adjunctive therapy for chronic stable angina: a randomized clinical trial. JAMA Intern Med. 2019;179:1388-1397.
489. Zheng H, Li J, Li Y, Zhao L, Wu X, Chen J, et al. Acupuncture for patients with mild hypertension: a randomized controlled trial. J Clin Hypertens (Greenwich). 2019;21:412-420.
490. Chen L, Deng H, Houle T, Zhang Y, Ahmed S, Zhang V, et al. A randomized trial to assess the immediate impact of acupuncture on quantitative sensory testing, pain, and functional status. Pain. 2019;160:2456-2463.
491. Kazemi A H, Wang W, Wang Y, et al. Therapeutic effects of acupuncture on blood glucose level among patients with type-2 diabetes mellitus: a randomized clinical trial. [Journal of Traditional Chinese Medical Sciences](https://kns.cnki.net/kns8/Navi?DBCode=CJFD&BaseID=ZYKZ)（English）. 2019; 6:7.
492. Yeung WF, Chung KF, Zhang ZJ, Zhang SP, Chan WC, Ng RM, et al. Electroacupuncture for tapering off long-term benzodiazepine use: a randomized controlled trial. J Psychiatr Res. 2019;109:59-67.
493. Garcia MK, Meng Z, Rosenthal DI, Shen Y, Chambers M, Yang P, et al. Effect of true and sham acupuncture on radiation-induced xerostomia among patients with head and neck cancer: a randomized clinical trial. JAMA Netw Open. 2019;2:e1916910.
494. Lv ZT, Shen LL, Zhu B, Zhang ZQ, Ma CY, Huang GF, et al. Effects of intensity of electroacupuncture on chronic pain in patients with knee osteoarthritis: a randomized controlled trial. Arthritis Res Ther. 2019;21:120.
495. Zheng Z, Bai L, O'Loughlan M, Li CG, Xue CC. Does electroacupuncture have different effects on peripheral and central sensitization in humans: a randomized controlled study. Front Integr Neurosci. 2019;13:61.
496. Zheng Z, Gibson S, Helme RD, Wang Y, Lu DS, Arnold C, et al. Effects of electroacupuncture on opioid consumption in patients with chronic musculoskeletal pain: a multicenter randomized controlled trial. Pain Med. 2019;20:397-410.
497. Kusuma AC, Oktari N, Mihardja H, Srilestari A, Simadibrata CL, Hestiantoro A, et al. Electroacupuncture enhances number of mature oocytes and fertility rates for in vitro fertilization. Med Acupunct. 2019;31:289-297.
498. Varejão CDS, Santo FHDE. Laser acupuncture for relieving nausea and vomiting in pediatric patients undergoing chemotherapy: a single-blind randomized clinical trial. J Pediatr Oncol Nurs. 2019;36:44-54.
499. Wu HC, Zhang JW, Sun ZG, Xiang S, Qiao Y, Lian F. Effects of electroacupuncture on expression of PI3K/Akt/Foxo3a in granulosa cells from women with Shen (Kidney) deficiency syndrome undergoing in vitro fertilization-embryo transfer. Chin J Integr Med. 2019;25:252-258.
500. Sg A, Hl B, Jg A, et al. Effect of transcutaneous electrical acupoint stimulation on gastrointestinal function recovery after laparoscopic radical gastrectomy – a randomized controlled trial - ScienceDirect. European Journal of Integrative Medicine. 2019; 26:11-17.
501. Mak AD, Chung VCH, Yuen SY, Tse YK, Wong SYS, Ju Y, et al. Noneffectiveness of electroacupuncture for comorbid generalized anxiety disorder and irritable bowel syndrome. J Gastroenterol Hepatol. 2019;34:1736-1742.
502. Smith CA, de Lacey S, Chapman M, Ratcliffe J, Norman RJ, Johnson NP, et al. The effects of acupuncture on the secondary outcomes of anxiety and quality of life for women undergoing IVF: a randomized controlled trial. Acta Obstet Gynecol Scand. 2019;98:460-469.
503. Shuai Z, Li X, Tang X, Lian F, Sun Z. Transcutaneous electrical acupuncture point stimulation improves pregnancy outcomes in patients with recurrent implantation failure undergoing in vitro fertilisation and embryo transfer: a prospective, randomised trial. Acupunct Med. 2019;37:33-39.
504. Chi YL, Zhang WL, Yang F, Su F, Zhou YK. Transcutaneous electrical acupoint stimulation for improving postoperative recovery, reducing stress and inflammatory responses in elderly patient undergoing knee surgery. Am J Chin Med. 2019;47:1445-1458.
505. Albooghobeish, Masoumeh, Ahmad Reza Mohtadi, Vahid Saidkhani, Golamreza Khataminia, Habib Fallah, et al. Comparative effects of the stimulation of BL10, BL11, and GB34 acupuncture points with P6 point using a low-level laser on the prevention of vomiting after strabismus surgery: a randomized, double-Blind, controlled clinical trial. Iranian Red Crescent Medical Journal. 2019.
506. Gao J, Zhao C, Jiang W, Zheng B, He Y. Effect of acupuncture on cognitive function and quality of life in patients with idiopathic trigeminal neuralgia. J Nerv Ment Dis. 2019;207:171-174.
507. Huang Z, Liu S, Zhou J, Yao Q, Liu Z. Efficacy and safety of acupuncture for chronic discogenic sciatica, a randomized controlled sham acupuncture trial. Pain Med. 2019;20:2303-2310.
508. Juan CW, Chang MH, Lin TH, Hwang KL, Fu TC, Shih PH, et al. Laser acupuncture for carpal tunnel syndrome: a single-blinded controlled study. J Altern Complement Med. 2019;25:1035-1043.
509. Feng B, Zhang Y, Luo LY, Wu JY, Yang SJ, Zhang N, et al. Transcutaneous electrical acupoint stimulation for post-traumatic stress disorder: assessor-blinded, randomized controlled study. Psychiatry Clin Neurosci. 2019;73:179-186.
510. Yu Y, Sha SB, Zhang B, Guan Q, Liang M, Zhao LG, et al. Effects and mechanism of action of transcutaneous electrical acupuncture point stimulation in patients with abnormal semen parameters. Acupunct Med. 2019;37:25-32.
511. Horta D, Lira A, Sanchez-Lloansi M, Villoria A, Teggiachi M, García-Rojo D, et al. A Prospective pilot randomized study: electroacupuncture vs. sham procedure for the treatment of fatigue in patients with quiescent inflammatory bowel disease. Inflamm Bowel Dis. 2020;26:484-492.
512. Liu J, Chen H, Wu D, Wei R, Lv C, Dong J, et al. Ameliorating effects of transcutaneous electrical acustimulation at Neiguan (PC6) and Zusanli (ST36) acupoints combined with adaptive biofeedback training on functional outlet obstruction constipation. Evid Based Complement Alternat Med. 2020;2020:8798974.
513. Yu S, Ortiz A, Gollub RL, Wilson G, Gerber J, Park J, et al. Acupuncture treatment modulates the connectivity of key regions of the descending pain modulation and reward systems in patients with chronic low back pain. J Clin Med. 2020;9:1719.
514. Kuzucu I, Karaca O. Acupuncture treatment in patients with chronic subjective tinnitus: A prospective, randomized study. Med Acupunct. 2020;32:24-28.
515. Barnard A, Jansen V, Swindells MG, Arundell M, Burke FD. A randomized controlled trial of real versus sham acupuncture for basal thumb joint arthritis. J Hand Surg Eur Vol. 2020;45:488-494.
516. Lin ZX, Chan NHT, Kwan YK, Chan YT, Zhang H, Tam KS, et al. A randomized controlled trial to assess the effectiveness and safety of acupuncture for overactive bladder: a study in Hong Kong population. Chin Med. 2020;15:108.
517. Gadau M, Zhang SP, Wang FC, Liguori S, Li WH, Liu WH, et al. A multi-center international study of acupuncture for lateral elbow pain - results of a randomized controlled trial. Eur J Pain. 2020;24:1458-1470.
518. Qin Z, Ding Y, Xu C, Kwong JSW, Ji Y, et al. Acupuncture vs noninsertive sham acupuncture in aging patients with degenerative lumbar spinal stenosis: a randomized controlled trial. Am J Med. 2020;133:500-507.e20.
519. Madani A, Ahrari F, Fallahrastegar A, Daghestani N. A randomized clinical trial comparing the efficacy of low-level laser therapy (LLLT) and laser acupuncture therapy (LAT) in patients with temporomandibular disorders. Lasers Med Sci. 2020;35:181-192.
520. Yu X, Zhang F, Chen B. The effect of TEAS on the quality of early recovery in patients undergoing gynecological laparoscopic surgery: a prospective, randomized, placebo-controlled trial. Trials. 2020;21:43.
521. Bastani, Fatemeh F, Seyedeh HS, Soudabeh KA, Forozan M, Bahareh K, et al. The effect of simultaneous application of transcutaneous electrical nerve stimulation (TENS) on specific lumbar and acupuncture points on labour pain relief: a randomised clinical trial. Journal of Clinical and Diagnostic Research. 2020.
522. Yin X, Li W, Wu H, Dong B, Ma J, Li S, et al. Efficacy of electroacupuncture on treating depression-related insomnia: a randomized controlled trial. Nat Sci Sleep. 2020;12:497-508.
523. Yang JW, Wang LQ, Zou X, Yan SY, Wang Y, Zhao JJ, et al. Effect of acupuncture for postprandial distress syndrome: a randomized clinical trial. Ann Intern Med. 2020;172:777-785.
524. Li S, Wang Z, Wu H, Yue H, Yin P, Zhang W, et al. Electroacupuncture versus sham acupuncture for perimenopausal insomnia: a randomized controlled clinical trial. Nat Sci Sleep. 2020;12:1201-1213.
525. Xiong W, Zhao CM, An LX, Xie SN, Jia CR. Efficacy of acupuncture combined with local anesthesia in ischemic stroke patients with carotid artery stenting: a prospective randomized trial. Chin J Integr Med. 2020;26:609-616.
526. Eberl S, Monteiro de Olivera N, Bourne D, Streitberger K, Fockens P, Hollmann MW, et al. Effect of electroacupuncture on sedation requirements during colonoscopy: a prospective placebo-controlled randomised trial. Acupunct Med. 2020;38:131-139.
527. Kong JT, Puetz C, Tian L, Haynes I, Lee E, Stafford RS, et al. Effect of electroacupuncture vs sham treatment on change in pain severity among adults with chronic low back pain: a randomized clinical trial. JAMA Netw Open. 2020;3:e2022787.
528. Surapaty IA, Simadibrata C, Rejeki ES, Mangunatmadja I. Laser acupuncture effects on speech and social interaction in patients with autism spectrum disorder. Med Acupunct. 2020;32:300-309.
529. Kholoosy L, Elyaspour D, Akhgari MR, Razzaghi Z, Khodamardi Z, Bayat M. Evaluation of the therapeutic effect of low level laser in controlling low back pain: a randomized controlled trial. J Lasers Med Sci. 2020;11:120-125.
530. Liao FY, Lin CL, Lo SF, Chang CC, Liao WY, Chou LW. Efficacy of acupoints dual-frequency low-level laser therapy on knee osteoarthritis. Evid Based Complement Alternat Med. 2020;2020:6979105.
531. Lee B, Kim BK, Kim HJ, Jung IC, Kim AR, Park HJ, et al. Efficacy and safety of electroacupuncture for insomnia disorder: a multicenter, randomized, assessor-blinded, controlled trial. Nat Sci Sleep. 2020;12:1145-1159.
532. Li QW, Yu MW, Wang XM, Yang GW, Wang H, Zhang CX, et al. Efficacy of acupuncture in the prevention and treatment of chemotherapy-induced nausea and vomiting in patients with advanced cancer: a multi-center, single-blind, randomized, sham-controlled clinical research. Chin Med. 20203;15:57.
533. Zhao F, Wang Z, Ye C, Liu J. Effect of transcutaneous electrical acupoint stimulation on one-lung ventilation-induced lung injury in patients undergoing esophageal cancer operation. Evid Based Complement Alternat Med. 2020; doi: 10.1155/2020/9018701.
534. Silva MVFP, Lustosa TC, Arai VJ, Couto PTLG, Lira MPF, Lins-Filho OL, et al. Effects of acupuncture on obstructive sleep apnea severity, blood pressure control and quality of life in patients with hypertension: a randomized controlled trial. J Sleep Res. 2020;29:e12954.
535. Sebayang RG, Aditya C, Abdurrohim K, Lauwrence B, Mihardja H, Kresnawan T, et al. Effects of laser acupuncture and dietary intervention on key obesity parameters. Med Acupunct. 2020;32:108-115.
536. Li M, Xu F, Liu M, Li Y, Zheng J, Zhu Y, et al. Effects and mechanisms of transcutaneous electrical acustimulation on postoperative recovery after elective cesarean section. Neuromodulation. 2020;23:838-846.
537. Chen J, Zhang Y, Li X, Wan Y, Ji X, Wang W, et al. Efficacy of transcutaneous electrical acupoint stimulation combined with general anesthesia for sedation and postoperative analgesia in minimally invasive lung cancer surgery: a randomized, double-blind, placebo-controlled trial. Thorac Cancer. 2020;11:928-934.
538. Yang JW, Wang LQ, Zou X, Yan SY, Wang Y, Zhao JJ, et al. Effect of acupuncture for postprandial distress syndrome: a randomized clinical trial. Ann Intern Med. 2020;172:777-785.
539. Song Y, Xue X, Han H, Li C, Jian J, Yuan W, et al. Efficacy of transcutaneous electrical acupoint stimulation combined with diazepam for acute alcohol withdrawal syndrome: a double-blind randomized sham-controlled trial. J Int Med Res. 2020; doi: 10.1177/0300060520910052.
540. Xu S, Yu L, Luo X, Wang M, Chen G, Zhang Q, et al. Manual acupuncture versus sham acupuncture and usual care for prophylaxis of episodic migraine without aura: multicentre, randomised clinical trial. BMJ. 2020;368:m697.
541. Putri DE, Srilestari A, Abdurrohim K, Mangunatmadja I, Wahyuni LK. The effect of laser acupuncture on spasticity in children with spastic cerebral palsy. J Acupunct Meridian Stud. 2020;13:152-156.
542. Zhang L, Tang Y, Hui R, Zheng H, Deng Y, Shi Y, et al. The effects of active acupuncture and placebo acupuncture on insomnia patients: a randomized controlled trial. Psychol Health Med. 2020;25:1201-1215.
543. Lo MY, Wu CH, Luh JJ, Wang TG, Fu LC, Lin JG, et al. The effect of electroacupuncture merged with rehabilitation for frozen shoulder syndrome: a single-blind randomized sham-acupuncture controlled study. J Formos Med Assoc. 2020;119:81-88.
544. Tian L, Feng X, Zhang R, Wang S, Li R, Kong R, et al. Pain relief during oocyte retrieval by transcutaneous electrical acupoint stimulation: a single-blinded, randomized, controlled multicenter trial. Evid Based Complement Alternat Med. 2020; doi: 10.1155/2020/3285648.
545. Hou M, Wang X, Yu J, Fu S, Yang F, Li Z, et al. The Effect of electroacupuncture on dynamic balance during stair climbing for elderly patients with knee osteoarthritis. Evid Based Complement Alternat Med. 2020; doi: 10.1155/2020/3563584.
546. Yu S, Xie M, Liu S, Guo X, Tian J, Wei W, et al. Resting-state functional connectivity patterns predict acupuncture treatment response in primary dysmenorrhea. Front Neurosci. 2020;14:559191.
547. Zhu Y, Li X, Ma J, Xu W, Li M, Gong Y, et al. Transcutaneous electrical acustimulation improves gastrointestinal disturbances induced by transcatheter arterial chemoembolization in patients with liver cancers. Neuromodulation. 2020;23:1180-1188.
548. Gülten A, Banu EE. The efficacy of acupuncture adding to standard postoperative care in patients undergoing laparoscopic cholecystectomy: a randomized controlled trial. Revista Internacional de Acupuntura. 2020; doi:10.1016/j.acu.2020.06.002.
549. Feiyi Z, Yan X, Li-ping Y, et al. Manual acupuncture for patients with major depressive disorder and comorbid insomnia: Evidence from polysomnography and serum biomarkers. World Journal of Acupuncture - Moxibustion. 2020;30:5-12.
550. Xu X, Zhang M, Wu X, Xu SB, Wang W, Zheng CH, et al. Efficacy of electro-acupuncture in treatment of functional constipation: a randomized controlled trial. Curr Med Sci. 2020;40:363-371.
551. Bao T, Patil S, Chen C, Zhi IW, Li QS, Piulson L, et al. Effect of acupuncture vs sham procedure on chemotherapy-induced peripheral neuropathy symptoms: a randomized clinical trial. JAMA Netw Open. 2020;3:e200681.
552. Li M, Xu F, Liu M, Li Y, Zheng J, Zhu Y, et al. Effects and mechanisms of transcutaneous electrical acustimulation on postoperative recovery after elective cesarean section. Neuromodulation. 2020;23:838-846.
553. Zhengrong X, Zecheng D, Dou X, Wenying Shi, Feng Z,Yuanyuan, T, et al. Acupuncture for chronic prostatitis: a randomized controlled trial.World Journal of Acupuncture - Moxibustion. 2022;32,204-207.
554. Li C, Li T, Ma X, et al. A randomized clinical study on acupuncture therapy for relieving sciatica caused by lumbar disc herniation. OMICS International. 2021; doi:10.36468/PHARMACEUTICAL-SCIENCES.SPL.284.
555. Sabbagh GA, Rezaei AA, Farahmand SK, Dadgarmoghaddam M, Ghorani V, Rezaei S, et al. Additive effects of acupuncture in alleviating anxiety: A double-blind, three-arm, randomized clinical trial. Complement Ther Clin Pract. 2021;45:101466.
556. Tu CH, Lee YC, Chen YY, Chen CM, Lu WC, Chen YH, et al. Acupuncture treatment associated with functional connectivity changes in primary dysmenorrhea: a resting state fMRI Study. J Clin Med. 2021;10:4731.
557. Ebadi S, Alishahi V, Ahadi T, Raissi GR, Khodabandeh M, Haqiqatshenas H, et al. Acupuncture-like versus conventional transcutaneous electrical nerve stimulation in the management of active myofascial trigger points: a randomized controlled trial. J Bodyw Mov Ther. 2021;28:483-488.
558. Zhang J, Lyu T, Yang Y, Wang Y, Zheng Y, Qu S, et al. Acupuncture at LR3 and KI3 shows a control effect on essential hypertension and targeted action on cerebral regions related to blood pressure regulation: a resting state functional magnetic resonance imaging study. Acupunct Med. 2021;39:53-63.
559. Chang WH, Tu LW, Pei YC, Chen CK, Wang SH, Wong AM. Comparison of the effects between lasers applied to myofascial trigger points and to classical acupoints for patients with cervical myofascial pain syndrome. Biomed J. 2021;44:739-747.
560. Lv JQ, Li PC, Zhou L, Tang WF, Li N. Acupuncture at the P6 acupoint to prevent postoperative pain after craniotomy: a randomized, placebo-controlled study. Evid Based Complement Alternat Med. 2021;2021:6619855.
561. Ismail AMA, El-Azeim ASA. Short-term intraocular pressure response to the combined effect of transcutaneous electrical nerve stimulation over acupoint (Acu-TENS) and yoga ocular exercise in type 2 diabetic patients with primary open-angle glaucoma: a randomized controlled trial. J Acupunct Meridian Stud. 2021;14:193-199.
562. Bao T, Baser R, Chen C, Weitzman M, Zhang YL, Seluzicki C, et al. Health-related quality of life in cancer survivors with chemotherapy-induced peripheral neuropathy: a randomized clinical trial. Oncologist. 2021;26:e2070-e2078.
563. Zhao L, Cheng K, Wu F, Du J, Chen Y, Tan MT, et al. Effect of laser moxibustion for knee osteoarthritis: a multisite, double-blind randomized controlled trial. J Rheumatol. 2021;48:924-932.
564. Meyer-Hamme G, Friedemann T, Greten J, Gerloff C, Schroeder S. Electrophysiologically verified effects of acupuncture on diabetic peripheral neuropathy in type 2 diabetes: the randomized, partially double-blinded, controlled ACUDIN trial. J Diabetes. 2021;13:469-481.
565. Rangel CRG, Pinheiro SL. Laser acupuncture and intravascular laser irradiation of blood for management of pediatric dental anxiety. J Oral Sci. 2021;63:355-357.
566. Huang L, Yin X, Li W, Cao Y, Chen Y, Lao L, et al. Effects of acupuncture on vascular cognitive impairment with no dementia: a randomized controlled trial. J Alzheimers Dis. 2021;81:1391-1401.
567. Wang C, Xu WL, Li GW, Fu C, Li JJ, Wang J, et al. Impact of acupuncture on sleep and comorbid symptoms for chronic insomnia: a randomized clinical trial. Nat Sci Sleep. 2021;13:1807-1822.
568. Park JG, Lee H, Yeom M, Chae Y, Park HJ, Kim K. Effect of acupuncture treatment in patients with mild to moderate atopic dermatitis: a randomized, participant- and assessor-blind sham-controlled trial. BMC Complement Med Ther. 2021;21:132.
569. Ton G, Lee LW, Ho WC, Tu CH, Chen YH, Lee YC. Effects of laser acupuncture therapy for patients with inadequate recovery from bell's palsy: preliminary results from randomized, double-blind, sham-controlled study. J Lasers Med Sci. 2021;12:e70.
570. Hu WL, Yu HJ, Pan LY, Wu PC, Pan CC, Kuo CE, et al. Laser acupuncture improves tear film stability in patients with dry eye disease: a two-center randomized-controlled trial. J Altern Complement Med. 2021;27:579-587.
571. Yu WZ, Huang CM, Ng HP, Lee YC. Distal acupoints outperform proximal acupoints in treating knee osteoarthritis: a randomized controlled trial. Evid Based Complement Alternat Med. 2021;2021:4827123.
572. Lynning M, Hanehøj K, Westergaard K, Kjær Ersbøll A, Claesson MH, Boesen F, et al. Effect of acupuncture on cytokine levels in persons with multiple sclerosis: a randomized controlled trial. J Altern Complement Med. 2021;27:832-840.
573. Chen Y, Gong Y, Huai X, Gu X, Su D, Yu W, et al. Effects of transcutaneous electrical acupuncture point stimulation on peripheral capillary oxygen saturation in elderly patients undergoing colonoscopy with sedation: a prospective randomized controlled trial. Acupunct Med. 2021;39:292-298.
574. Zhai ZJ, Liu JE, Lei LL, Wang SY. Effects of transcutaneous electrical acupoint stimulation on ovarian responses and pregnancy outcomes in patients undergoing IVF-ET: a randomized controlled trial. Chin J Integr Med. 2022;28:434-439.
575. Ao L, Shi J, Bai Y, Zhang S, Gan J. Effects of transcutaneous electrical acupoint stimulation on perioperative immune function and postoperative analgesia in patients undergoing radical mastectomy: a randomized controlled trial. Exp Ther Med. 2021;21:184.
576. Sui M, Jiang N, Yan L, Liu J, Luo B, Zhang C, et al. Effect of electroacupuncture on shoulder subluxation in poststroke patients with hemiplegic shoulder pain: a sham-controlled study using multidimensional musculoskeletal ultrasound assessment. Pain Res Manag. 2021;2021:5329881.
577. Wang Y, Xu J, Zhang Q, Zhang Q, Yang Y, Wei W, et al. Immediate analgesic effect of acupuncture in patients with primary dysmenorrhea: A fMRI study. Front Neurosci. 2021;15:647667.
578. Teoh AYB, Chong CCN, Leung WW, Chan SKC, Tse YK, Ng EKW, et al. Electroacupuncture to reduce sedative and analgesic demands during endoscopic ultrasonography: a prospective, randomised, double-blind, sham-controlled study (abridged secondary publication). Hong Kong Med J. 2021;27 Suppl 2:4-7.
579. Zhao QY, Sun Y, Zhou J, Gao YL, Ma GZ, Hu ZH, et al. Effectiveness of herb-partitioned moxibustion combined with electroacupuncture on polycystic ovary syndrome in patients with symptom pattern of kidney deficiency and phlegm-dampne. J Tradit Chin Med. 2021;41:985-993.
580. Maheshkumar K, Deenadayalan B, Akila A, et al. Effectiveness of Taichong (LR3) acupuncture point on blood Pressure in patients with hypertension: a randomized sham control trial. Advances in Integrative Medicine. 2020; doi:10.1016/j.aimed.2020.05.002.
581. Que B, Tu Q, Shi J, Wan Z, Li Y, Zhou R, et al. Effects of transcutaneous electrical acupoint stimulation on systemic inflammatory response syndrome of patients after percutaneous nephrolithotomy: a randomized controlled trial. Evid Based Complement Alternat Med. 2021;2021:5909956.
582. Kim KW, Shin WC, Choi MS, Cho JH, Park HJ, Yoo HH, et al. Effects of acupuncture on anthropometric and serum metabolic parameters in premenopausal overweight and obese women: a randomized, patient- and assessor-blind, sham-controlled clinical trial. Acupunct Med. 2021;39:30-40.
583. Lu Z, Wang Q, Sun X, Zhang W, Min S, Zhang J, et al. Transcutaneous electrical acupoint stimulation before surgery reduces chronic pain after mastectomy: a randomized clinical trial. J Clin Anesth. 2021;74:110453.
584. Liu C, Zhao Y, Qin S, Wang X, Jiang Y, Wu W. Randomized controlled trial of acupuncture for anxiety and depression in patients with chronic insomnia. Ann Transl Med. 2021;9:1426.
585. Szmit M, Agrawal S, Goździk W, Kübler A, Agrawal A, Pruchnicki P, et al. Transcutaneous electrical acupoint stimulation reduces postoperative analgesic requirement in patients undergoing inguinal hernia repair: a randomized, placebo-controlled Study. J Clin Med. 2021;10:146.
586. Yeung WF, Yu BY, Yuen JW, Ho JYS, Chung KF, Zhang ZJ, et al. Semi-individualized acupuncture for insomnia disorder and oxidative stress: a randomized, double-blind, sham-controlled trial. Nat Sci Sleep. 2021;13:1195-1207.
587. Zheng Y, Jiang X, Gao Y, Yuan L, Wang X, Wu S, et al. Microbial profiles of patients with antipsychotic-related constipation treated with electroacupuncture. Front Med (Lausanne). 2021;8:737713.
588. Qiu G, Huang T, Lu Y, Zhang L, Zhao Y, Yuan Y, et al. Perioperative electroacupuncture can accelerate the recovery of gastrointestinal function in cancer patients undergoing pancreatectomy or gastrectomy: a randomized controlled Trial. Evid Based Complement Alternat Med. 2021;2021:5594263.
589. Gao W, Li W, Yan Y, Yang R, Zhang Y, Jin M, et al. Transcutaneous electrical acupoint stimulation applied in lower limbs decreases the incidence of paralytic ileus after colorectal surgery: a multicenter randomized controlled trial. Surgery. 2021;170:1618-1626.
590. Li WJ, Gao C, An LX, Ji YW, Xue FS, Du Y. Perioperative transcutaneous electrical acupoint stimulation for improving postoperative gastrointestinal function: a randomized controlled trial. J Integr Med. 2021;19:211-218.
591. Allameh F, Razzaghi M, Hosseini S, Barati M, Razzaghi Z, Salehi S, et al. The effect of laser acupuncture on semen parameters in infertile men with oligospermia: a randomized clinical trial. J Lasers Med Sci. 2021;12:e84.
592. Zhang Y, Wang Z, Du J, Liu J, Xu T, Wang X, et al. Regulatory effects of acupuncture on emotional disorders in patients with menstrual migraine without aura: a resting-state fMRI study. Front Neurosci. 2021;15:726505.
593. Xi L, Fang F, Yuan H, Wang D. Transcutaneous electrical acupoint stimulation for postoperative cognitive dysfunction in geriatric patients with gastrointestinal tumor: a randomized controlled trial. Trials. 2021;22:563.
594. M Alwhaibi R, Mahmoud NF, M Zakaria H, M Ragab W, Al Awaji NN, Y Elzanaty M, et al. Therapeutic efficacy of transcutaneous electrical nerve stimulation acupoints on motor and neural recovery of the affected upper extremity in chronic stroke: a sham-controlled randomized clinical trial. Healthcare (Basel). 2021;9:614.
595. Xuan JL, Zhu YW, Xu WH, Zhao H, Chen JDZ, Wu GJ, et al. Integrative effects of transcutaneous electrical acustimulation on abdominal pain, gastrointestinal motility, and inflammation in patients with early-stage acute pancreatitis. Neurogastroenterol Motil. 2022;34:e14249.
596. Hu P, Sun K, Li H, Qi X, Gong J, Zhang Y, et al. Transcutaneous electrical acustimulation improved the quality of life in patients with diarrhea-irritable bowel syndrome. Neuromodulation. 2022;25:1165-1172.
597. Abad-Constantino R, Caro-Chang L M, Gatmaitan-Dumlao J, et al. Acupuncture as an adjunct to standard therapy for pruritus in patients with atopic dermatitis: a patient- and assessor-blinded, randomized, placebo-controlled trial. University of the Philippines Manila. 2021; doi:10.47895/AMP.V55I5.3226.
598. Cai W, Ma W, Li YJ, Wang GT, Yang H, Shen WD. Efficacy and safety of electroacupuncture for post-stroke depression: a randomized controlled trial. Acupunct Med. 2022;40:434-442.
599. Hui H, Shi-ke Z, Wei-bin G, et al. Electroacupuncture for postpartum urinary retention: a randomized controlled study. World Journal of Acupuncture - Moxibustion. 2021; doi:[10.1016/j.wjam.2021.05.012](https://www.x-mol.com/paperRedirect/1394752292550852608).
600. Mawla I, Ichesco E, Zöllner HJ, Edden RAE, Chenevert T, Buchtel H, et al. Greater somatosensory afference with acupuncture increases primary somatosensory connectivity and alleviates fibromyalgia pain via insular γ-Aminobutyric acid: a randomized neuroimaging trial. Arthritis Rheumatol. 2021;73:1318-1328.
601. Zhai ZJ, Liu JE, Lei LL, Wang SY. Effects of transcutaneous electrical acupoint stimulation on ovarian responses and pregnancy outcomes in patients undergoing IVF-ET: a randomized controlled trial. Chin J Integr Med. 2022;28:434-439.
602. Zhang B, Hu Y, Shi X, Li W, Zeng X, Liu F, et al. Integrative effects and vagal mechanisms of transcutaneous electrical acustimulation on gastroesophageal motility in patients with gastroesophageal reflux disease. Am J Gastroenterol. 2021;116:1495-1505.
603. Szmit M, Agrawal S, Goździk W, Kübler A, Agrawal A, Pruchnicki P, et al. Transcutaneous electrical acupoint stimulation reduces postoperative analgesic requirement in patients undergoing inguinal hernia repair: a randomized, placebo-controlled study. J Clin Med. 2021;10:146.
604. D'Alessandro EG, da Silva AV, Cecatto RB, de Brito CMM, Azevedo RS, Lin CA. Acupuncture for climacteric-like symptoms in breast cancer improves sleep, mental and emotional health: a randomized trial. Med Acupunct. 2022;34:58-65.
605. Bao C, Wu L, Wang D, Chen L, Jin X, Shi Y, et al. Acupuncture improves the symptoms, intestinal microbiota, and inflammation of patients with mild to moderate Crohn's disease: a randomized controlled trial. EClinicalMedicine. 2022;45:101300.
606. Wen Q, Hu M, Lai M, Li J, Hu Z, Quan K, et al. Effect of acupuncture and metformin on insulin sensitivity in women with polycystic ovary syndrome and insulin resistance: a three-armed randomized controlled trial. Hum Reprod. 2022;37:542-552.
607. Brase A, Brauchle D, Kermad A, Volk T, Morinello E, Gottschling S, et al. Postoperative pain therapy with laser acupuncture after cesarean section under spinal anesthesia: a double-blinded, randomized, placebo-controlled trial. Complement Med Res. 2022;29:235-241. English.
608. Drews T, Hummel T, Rochlitzer B, Hauswald B, Hähner A. Acupuncture is associated with a positive effect on odour discrimination in patients with postinfectious smell loss-a controlled prospective study. Eur Arch Otorhinolaryngol. 2022;279:1329-1334.
609. Pan W, Li FX, Wang Q, Huang ZQ, Yan YM, Zhao L, et al. A randomized sham-controlled trial of manual acupuncture for infertile women with polycystic ovary syndrome. Integr Med Res. 2022;11:100830.
