# Supplementary material for: Descriptions of sham acupuncture in randomised controlled trials: a critical review of the literature
Source: BMC Complement Med Ther. 2023 May 30;23:173. doi: 10.1186/s12906-023-04007-7 (PMC10227975; doi:10.1186/s12906-023-04007-7)
Supplement: Supplementary file 4 — Supplementary Material 4 [file 12906_2023_4007_MOESM4_ESM.docx]

| **Table S1** Sham laser acupuncture and sham TENS/TEAS related items recommended in both STRICTA 2010 and TIDieR-Placebo | | | |
| --- | --- | --- | --- |
| **Items in STRICTA 2010** | | **Items in TIDieR-Placebo** | **Proportion** |
| Acupuncture rationale | (1a) Style of acupuncture (eg, Traditional Chinese Medicine, Japanese, Korean, Western medical, Five Element, ear acupuncture, etc) |  | NR |
|  | (1b) Reasoning for treatment provided, based on historical context, literature sources and/or consensus methods, with references where appropriate | 2.Why: Describe any rationale, theory, or goal of the elements essential to the placebo/ sham intervention | 44.0% |
|  | (1c) Extent to which treatment was varied | 9.Tailoring: If the placebo/sham intervention was planned to be personalised, titrated, or adapted, then describe what, why, when, and how | 3.6% |
| Details of needling | 2a) Number of needle insertions per subject per session (mean and range where relevant) | 4.What (Procedure) :Describe each of the procedures, activities, and/or processes used in the placebo/sham intervention, including any enabling or support activities | 51.8% |
|  | 2b) Names (or location if no standard name) of points used (uni/bilateral) |  | 87.5% |
|  | 2c) Depth of insertion, based on a specified unit of measurement, or on a particular tissue level |  | NR |
|  | 2d) Response sought (e.g. de qi or muscle twitch response) |  | NR |
|  | 2e) Needle stimulation (e.g. manual, electrical) |  | 72.6% |
|  | 2f) Needle retention time |  | 66.1% |
|  | 2g) Needle type (diameter, length, and manufacturer or material) | 3.What (materials): Describe any physical or informational materials used in the placebo/sham intervention, including those provided to participants or used in intervention delivery or in training of intervention providers. Provide information on where the materials can be accessed (such as an online appendix, URL) | 95.2%(type) |
|  |  |  | 30.4%(size) |
|  |  |  | 8.3%([assisting tools](javascript:;)) |
|  |  |  | 36.3%  (manufacturer) |
| Treatment regimen | (3a) Number of treatment sessions | 8.When and how much: Describe the number of times the placebo/sham intervention was delivered and over what period of time, including the number of sessions, their schedule, and their duration, intensity, or dose. If relevant, include the duration of the pre- and postrandomisation consultations | 76.8% |
|  | (3b) Frequency and duration of treatment sessions |  | 77.4% |
|  |  |  | 77.4% |
| NR=Not relevant | | | |

| **Table S2** Items about sham laser acupuncture and sham TENS/TEAS only recommended in TIDieR-Placebo | |
| --- | --- |
| **Items** | **Proportion** |
| 1. Brief name: Provide the name or a phrase that describes the placebo/sham intervention | 100% |
| 5.Who provided: For each category of placebo/sham intervention provider (such as psychologist, nursing assistant), describe their expertise, background, and any specific training given | 11.3%(Work experience) |
|  | 19.0%(Educational background) |
|  | 9.5%(Training) |
|  | 25.6%(Profession) |
| 6.How: Describe the modes of delivery (such as face to face or by some other mechanism, such as internet or telephone) of the intervention and whether it was provided individually or in a group | 9.5% |
| 7.Where: Describe the type(s) of locations(s) and settings where the placebo/sham intervention occurred, including any necessary infrastructure or relevant features | 0.6% |
| 10.Modifications: If the placebo/sham intervention was modified during the course of the study, describe the changes (what, why, when, and how) | 0 |
| 11. How well (planned): If placebo/sham intervention adherence or fidelity was assessed, describe how and by whom, and if any strategies were used to maintain or improve fidelity, describe them | 0 |
| 12.How well (actual): If placebo/sham intervention adherence or fidelity was assessed,  describe the extent to which the intervention was delivered as planned | 0 |
| 13.Measuring the success of blinding: Was blinding measured, and if so, how, and what were the results of such measurement | 3.6% |

| **Table S3** Other items related to sham laser acupuncture and sham TENS/TEAS | | | |
| --- | --- | --- | --- |
| **Reporting items** | **Numerator** | **Denominator** | **Reporting proportion** |
| Model of electrical equipment | 72 | 168 | 42.9% |
| Intervention information offered to patients | 36 | 168 | 21.4% |
| Patient body position | 23 | 168 | 13.7% |
| Communication with participants before intervention | 23 | 168 | 13.7% |
| Disinfection | 15 | 168 | 8.9% |
| Communication with participants during intervention | 10 | 168 | 6.0% |
| Communication with participants after intervention | 10 | 168 | 3.0% |
| Information offered in informed consent | 2 | 168 | 1.2% |
| Operation before needle insertion(stimulation) | 1 | 168 | 0.6% |
| Operation after needle removing(stimulation) | 2 | 168 | 1.2% |
